# Differential Risks of Dementia, Depression, and Injury Among Common α-Blockers, with Tamsulosin as the Reference Drug: A Real-World Cohort Study in Men with Lower Urinary Tract Symptoms

**DOI:** 10.3390/jcm14238302

**Published:** 2025-11-22

**Authors:** Sunny Ssu-Yu Chen, Ya-Chuan Chang, Chia-Ying Yu, Tzuo-Yi Hsieh, Wen-Wei Sung

**Affiliations:** 1 School of Medicine, Chung Shan Medical University, No. 110, Section 1, Jianguo North Road, Taichung 40201, Taiwan; k95081@gmail.com (S.S.-Y.C.); or raptor7037@gmail.com (Y.-C.C.); or cyyu2015@gmail.com (C.-Y.Y.); 2 Institute of Neuroscience, National Yang Ming Chiao Tung University, Taipei 30010, Taiwan; 3 Department of Urology, Chung Shan Medical University Hospital, No. 110, Section 1, Jianguo North Road, Taichung 40201, Taiwan; 4 Institute of Medicine, Chung Shan Medical University, Taichung 40201, Taiwan

**Keywords:** α-blocker, benign prostatic hyperplasia, dementia, Alzheimer’s disease, depressive disorder

## Abstract

**Background/Objectives:** Although α-blockers are commonly used to treat lower urinary tract symptoms in men with benign prostatic hyperplasia (BPH), their differential neuropsychiatric safety profiles remain underexplored. Some studies have suggested an increased risk of cognitive decline, mood disorders, and falls, but the results remain inconclusive. **Methods:** We conducted a large retrospective cohort study using the TriNetX global network, identifying over 264,000 men treated with a single α-blocker between 2005 and 2023. Patients were grouped by α-blocker type—tamsulosin, doxazosin, terazosin, alfuzosin, silodosin, or prazosin—and matched 1:1 using propensity scores to adjust for demographic, clinical, and psychosocial variables. The primary outcomes were new-onset dementia, depression, and unintentional injuries, assessed at 1-, 3-, and 10-year intervals. The median follow-up duration was approximately 5.5 years, ranging from 1824 to 2200 days across cohorts, indicating balanced observation periods among the six α-blocker groups. **Results:** Among the included patients, tamsulosin (*n* = 213,690) and prazosin (n = 1184) were associated with significantly higher risks of neurodegenerative, psychiatric, and injury-related outcomes. Alfuzosin (n = 10,138) exhibited the most favorable safety profile across all endpoints. The findings remained robust in sensitivity analyses, excluding patients with prior malignancy or other α-blocker exposure. **Conclusions:** Substantial differences in long-term neuropsychiatric safety exist among α-blockers. Given its favorable profile, alfuzosin may be the preferred agent in patients at elevated risk of cognitive or psychiatric disorders. These findings highlight the need for individualized α-blocker selection and long-term pharmacovigilance in BPH management.

## 1. Background

α-Blockers are a vital class of clinical medications that play a key role in the management of both cardiovascular diseases [1] and benign prostatic hyperplasia (BPH) [2]. These agents exert their effects by inhibiting the contraction of vascular smooth muscles, thereby contributing to blood pressure control [1]. In addition, α-blockers act on the smooth muscle of the prostatic urethra, promoting urethral relaxation and improving urinary flow [3]. Currently, α-blockers are classified based on their receptor subtype selectivity. First-generation α-blockers bind non-selectively to all α1-adrenergic receptor subtypes (α1A, α1B, and α1D), while second-generation α-blockers are more prostate-specific, exhibiting either clinical uroselectivity or true α1A receptor selectivity—the predominant α-receptor subtype found in the prostate [4]. Among the commonly used agents, doxazosin and terazosin are classified as first-generation α-blockers, while alfuzosin, tamsulosin, and silodosin are considered second-generation agents [5]. Since the 1990s, the development of α-antagonists has significantly advanced the treatment of BPH and lower urinary tract symptoms, reshaping clinical practice in this domain [2].

In clinical practice, a wide range of α-blockers is available, and some have demonstrated symptom improvement in certain psychiatric disorders [6,7], underscoring their complex effects on neuropsychiatric conditions. Previous studies have explored the neurological and functional implications of α-blocker use. Several reports have suggested an association between α-blocker use and increased risks of dementia [8,9], depression [8,10,11], and unintentional injuries [12,13], while other studies have presented conflicting findings or found no significant associations [14,15]. Comparative analyses among different α-blockers have also been conducted [16,17,18]. These discrepancies may be attributed to methodological limitations, including heterogeneous medication use patterns, short follow-up durations, small cohort sizes, and inadequate adjustments for confounding factors, such as marital status and substance abuse. Such limitations may compromise the validity of the findings. In addition, recent clinical research has largely focused on the neuropsychiatric effects of 5α-reductase inhibitors (5ARIs) [19], often neglecting the potential concurrent use of various α-blockers, which could significantly influence patient outcomes.

In this study, we employ a retrospective cohort design to examine whether the use of α-blockers is associated with differential risks of dementia, depression, and unintentional injuries. By including a large patient population and rigorously excluding individuals who have used other α-blockers or 5ARIs, we aim to minimize confounding from concomitant drug exposure. Furthermore, we adjust for key factors, such as marital status [20,21] and substance use disorders [21], to reduce the effect of external influences on neuropsychiatric outcomes. Through this methodologically robust approach, we seek to provide a comprehensive assessment of the associations between α-blocker use and these conditions, offering valuable insights to inform clinical decision-making and medication management.

## 2. Methods

### 2.1. Data Source, Study Design, and Group Selection

This retrospective study utilized data from the TriNetX Global Collaborative Network (https://trinetx.com, accessed on 22 December 2024), a federated health research platform comprising large datasets from 146 healthcare organizations (HCOs) across all continents. Collectively, these HCOs provide access to data from over 275 million patients. The TriNetX database harmonizes data using standard terminologies, such as the International Classification of Diseases, Tenth Revision, Clinical Modification (ICD-10-CM), Anatomical Therapeutic Chemical (ATC) codes, and Logical Observation Identifiers Names and Codes, enabling efficient data indexing without the need for additional data wrangling.

**Figure 1:**
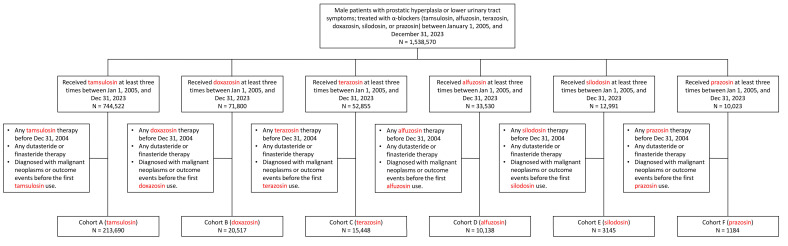
Cohort selection flow for the primary analysis, including participants with a history of other α-blocker use. This flow diagram illustrates the inclusion and exclusion process leading to the final study cohort. Patients with lower urinary tract symptoms who received α-blocker therapy were screened according to predefined criteria, and those meeting all eligibility requirements were included in the tamsulosin and comparator groups for the main analysis.

A flowchart illustrating the study design is presented in Figure 1. We initially included male patients diagnosed with prostatic hyperplasia or lower urinary tract symptoms who received treatment with α-blockers between 1 January 2005, and 31 December 2023. The patients were categorized into five groups based on the subsequent initiation of different α-blockers. The third α-blocker prescription was defined as the index event—the starting point of drug exposure—and patients with fewer than three α-blocker prescriptions were excluded. In addition, patients who had received any α-blocker therapy prior to 31 December 2004, were excluded from the analysis. For instance, in the tamsulosin group, patients who had used tamsulosin before this cutoff date were removed.

We also excluded patients who had used 5ARIs, including dutasteride and finasteride, due to previous studies suggesting their potential association with neuropsychiatric disorders [22]. Patients with a prior diagnosis of malignant neoplasms or any of the study outcomes before their first use of 5ARIs were also excluded. Drug prescriptions were identified using ATC codes, and disease diagnoses were defined using ICD-10-CM codes (Supplementary Table S1).

To assess the robustness of our findings, we conducted sensitivity analyses. First, we limited the cohorts to patients who had not used any other α-blockers and who had not used 5ARIs to reduce potential confounding. Second, we removed the exclusion criterion of prior malignant neoplasm diagnosis to evaluate whether a history of cancer affected the study outcomes.

### 2.2. Outcomes

The primary outcomes of interest in this study were categorized into three groups: neurodegenerative diseases, psychiatric disorders, and unintentional injuries. Each outcome was evaluated for risk at 1-, 3-, and 10-year intervals. Neurodegenerative diseases were identified using the following indicators: dementia (ICD-10-CM: F01–F03), mental disorders due to known physiological conditions (ICD-10-CM: F01–F09), Alzheimer’s disease (ICD-10-CM: G30), and other degenerative diseases of the nervous system (ICD-10-CM: G30–G32). Psychiatric disorders were assessed based on mood disorders (ICD-10-CM: F30–F39), depressive disorders (ICD-10-CM: F32–F33), and anxiety-related conditions, including anxiety, dissociative, stress-related, somatoform, and other nonpsychotic mental disorders (ICD-10-CM: F40–F48), with a specific focus on other anxiety disorders (ICD-10-CM: F41). Unintentional injuries were classified into head injuries (ICD-10-CM: S00–S09) and hip and thigh injuries (ICD-10-CM: S70–S79) (Supplementary Table S1).

### 2.3. Covariate Data

To minimize potential bias between the study groups, we performed 1:1 propensity score matching (PSM) using the TriNetX platform. The matching process accounted for a broad range of factors associated with neurodegenerative diseases and psychiatric disorders, including (a) demographic variables: age at index date, sex, race, and marital status; (b) comorbidities: diabetes mellitus, malnutrition, obesity, metabolic disorders, hypertensive diseases, cerebrovascular diseases, vascular diseases, and substance use disorders; and (c) laboratory data: body mass index. PSM was conducted prior to each analysis to ensure the robustness of the comparisons. Following matching, all covariates had *p*-values > 0.05 and standardized mean differences (SMDs) < 0.1, indicating good balance between groups.

### 2.4. Statistical Analysis

Statistical analyses were conducted using the TriNetX platform. Cox proportional hazard regression models were employed to estimate the hazard ratios (HRs) and 95% confidence intervals (CIs) to evaluate the associations between α-blocker use and study outcomes. The proportional hazards assumption was assessed using the generalized Schoenfeld test. All statistical tests were two-sided, and *p*-values < 0.05 were considered statistically significant.

## 3. Results

### 3.1. Baseline Characteristics of α-Blocker Users Before and After Matching

This study involved multiple cohort comparisons. A total of 213,690 patients using tamsulosin, 20,517 using doxazosin, 15,448 using terazosin, 10,138 using alfuzosin, 3145 using silodosin, and 1184 using prazosin met the inclusion criteria (Figure 1). Prior to matching, the mean age at the index event for patients in the tamsulosin group was 66.8 ± 11.9 years. The corresponding mean ages in the doxazosin, terazosin, alfuzosin, silodosin, and prazosin groups were 67.3 ± 11.7, 69.1 ± 11.5, 64.1 ± 11.6, 66.0 ± 11.6, and 60.8 ± 14.7 years, respectively. In this study, tamsulosin was compared with each of the five other α-blockers. After PSM, both comparison groups demonstrated balanced comorbidity profiles, with SMDs below 0.1. The detailed baseline characteristics for each group are provided in Supplementary Tables S2–S6.

### 3.2. Comparative Risk Analysis of α-Blockers for Neuropsychiatric Outcomes and Injuries

**Figure 2:**
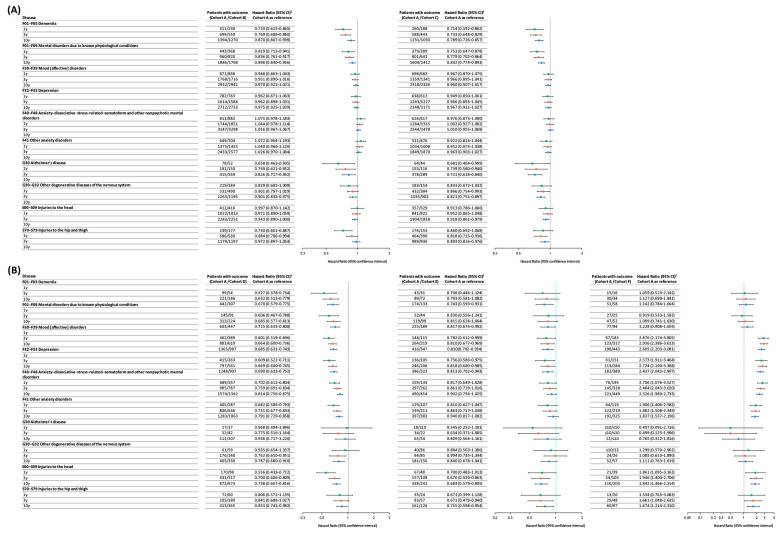
Hazard ratios for dementia, depression, and unintentional injury among cohorts that included participants with a history of other α-blocker use. This figure presents the hazard ratios and 95% confidence intervals for the primary outcomes—dementia, depression, and unintentional injury—comparing tamsulosin with (**A**) doxazosin and terazosin and (**B**) alfuzosin, silodosin, and prazosin in the main analysis cohort. Each panel shows the results for the different follow-up durations (1-, 3-, 5-, and 10-year analyses). The green, red and blue dot represents each disease’s hazard ratio of 1, 3 and 10 years.

Figure 2 presents the detailed results of the study. In the comparison between tamsulosin and doxazosin, doxazosin was associated with a significantly lower risk of neurodegenerative diseases. Statistically significant HRs (HRs [95% CI]) for dementia were observed at 1 year (0.729 [0.615–0.865]), 3 years (0.769 [0.688–0.860]), and 10 years (0.870 [0.807–0.939]). Higher risks for mental disorders due to known physiological conditions and Alzheimer’s disease were also observed in the tamsulosin group, with statistically significant differences. For other degenerative diseases, a lower risk was noted in the doxazosin group, although statistical significance was reached only at the 10-year follow-up.

Regarding psychiatric disorders, doxazosin users exhibited lower risks for mood and depressive disorders, but these differences were not statistically significant. For anxiety, dissociative, stress-related, somatoform, and other nonpsychotic mental disorders, as well as other anxiety disorders, the doxazosin group showed slightly higher risks, but none reached statistical significance. In terms of unintentional injuries, doxazosin was associated with significantly lower risks for hip and thigh injuries at 1 year (HR 0.730 [0.601–0.887]) and 3 years (HR 0.884 [0.786–0.994]); the difference at 10 years was not statistically significant. For head injuries, although the doxazosin group showed a lower risk at all time points, these differences were not statistically significant. Overall, doxazosin use was associated with a lower risk of neurodegenerative diseases, psychiatric disorders, and unintentional injuries compared with tamsulosin.

In the comparison between tamsulosin and terazosin, terazosin generally demonstrated lower risks across all outcome categories. This was particularly notable for neurodegenerative diseases in which terazosin was associated with significantly lower risks of dementia, mental disorders due to known physiological conditions, and Alzheimer’s disease at 1-, 3-, and 10-year follow-ups. For other degenerative diseases, significant reductions were observed at the 3- and 10-year marks. Although the differences in psychiatric disorder outcomes did not reach statistical significance, terazosin consistently showed lower risks. A similar trend was observed for unintentional injuries, with terazosin users experiencing generally lower risks.

In the comparison of tamsulosin versus alfuzosin, the differences were even more pronounced. Alfuzosin was associated with significantly lower risks for neurodegenerative diseases, psychiatric disorders, and unintentional injuries across multiple outcomes, suggesting a stronger protective effect than doxazosin or terazosin. When comparing tamsulosin with silodosin, lower risks were generally observed in the silodosin group across all three outcome categories, but the differences were less pronounced and less frequently statistically significant compared to other drug comparisons. Finally, in the comparison between tamsulosin and prazosin, prazosin was the only α-blocker associated with higher risks across all three major outcomes. Multiple categories demonstrated statistically significant increases, suggesting a potentially unfavorable risk profile in this context.

### 3.3. Sensitivity Analyses of Cohort Selection Criteria

We conducted sensitivity analyses to assess the robustness of our cohort selection. Prior to each sensitivity analysis, covariate matching was performed using the same methodology as in the primary analysis (Supplementary Tables S7–S16).

First, we excluded patients who had used other types of α-blockers (Supplementary Figures S1 and S2). The overall trends in the comparisons of the three primary outcomes remained largely consistent with those observed in the main analysis. Notably, several outcomes that were not statistically significant in the primary analysis reached statistical significance in this sensitivity analysis, such as the protective effects of doxazosin and terazosin on psychiatric disorders. We attribute these findings to the reduced influence of drug interactions, which may have allowed the specific protective effects of individual α-blockers to become more apparent.

Second, we modified the inclusion criteria by removing the restriction that excluded patients with a history of cancer (Supplementary Figures S3 and S4). The results remained broadly consistent with those of the primary analysis, with even more pronounced protective effects observed for doxazosin, terazosin, alfuzosin, and silodosin. This suggests that the effect of cancer on the measured outcomes may be less substantial than the effects of α-blocker use.

## 4. Discussion

This retrospective cohort study represents the largest analysis to date of patients treated with α-blockers, employing stringent inclusion criteria related to drug type and usage patterns to ensure data reliability. The results demonstrated that different α-blockers were associated with varying risks of developing dementia, depression, and unintentional injuries. Among the α-blockers evaluated, prazosin was linked to the highest risk of these outcomes, followed by tamsulosin. By contrast, alfuzosin was associated with the lowest risk and appeared to be the safest option in this study. These findings have important clinical implications, particularly for patients with comorbidities for whom selecting α-blockers with lower neuropsychiatric risk profiles may help reduce the likelihood of adverse outcomes.

Different α-blockers exhibit distinct pharmacokinetic properties, which may contribute to the observed variation in disease risk. In animal models, the activation of α1-adrenoceptors—particularly the α1A subtype—has been associated with enhanced cognitive function [22] and antidepressive effects [23]. In addition, reduced expression of α1A-adrenoceptors has been reported in the brains of patients with dementia [24]. Given the pivotal role of α-receptors in the brain, the potential effect of α-blockers on central nervous system (CNS) function has been the subject of considerable debate. More importantly, α-blockers differ in their ability to cross the blood–brain barrier (BBB). For example, tamsulosin has relatively limited BBB penetration [25], whereas alfuzosin, doxazosin, and terazosin exhibit greater lipophilicity [26], allowing for more substantial CNS access. These findings suggest that α-blockers may exert differential effects on CNS function, potentially influenced by their unique pharmacological characteristics.

Compared with previous studies, our study adopts a more rigorous methodology to minimize potential sources of bias. Earlier studies often involved complex patterns of medication use in which observed outcomes could be confounded by the effects of concomitant drugs, thereby compromising the validity of the findings. Furthermore, the neurological and psychiatric outcomes assessed in our study typically develop over a prolonged period [27], and the relatively short follow-up durations used in prior research may have contributed to the unstable or inconclusive results. In addition, key factors known to influence neurological and psychiatric conditions, such as marital status [20] and substance use disorders [28], were frequently overlooked, further increasing the risk of bias. By contrast, our study accounts for these confounding variables and separately evaluates the pharmacological properties of different α-blockers. We believe that these methodological improvements contribute to a more accurate assessment of the disease risks associated with various α-blockers and offer meaningful guidance for clinical decision-making.

Plausible neurobiological pathways link subtype selectivity and BBB permeability to cognitive outcomes. First, α1A receptors are enriched in hippocampal and prefrontal circuits, where they promote long-term potentiation, working memory, and attention [29]. Sufficient CNS exposure to α1 antagonists could blunt these processes [22]. Second, α1A receptors on astrocytes trigger Ca^2+^-dependent gliotransmission that supports engram stabilization and memory consolidation, which means that centrally penetrant antagonists may dampen this signaling [30,31,32]. Third, the α1-mediated control of cerebrovascular tone couples systemic blood pressure with regional cerebral perfusion. α-Blockers can precipitate orthostatic hypotension and transient cerebral hypoperfusion, as well as the states linked to cognitive decline in older adults [33,34]. Fourth, noradrenergic regulation of arousal via the locus coeruleus [35,36] implies that centrally acting agents could increase daytime sleepiness and reduce attentional resources [37]. Additional crosstalk with cholinergic transmission [38] and microglial reactivity [39,40] offers more routes through which α1 blockade could alter synaptic plasticity and neuroinflammation.

Drug-specific biology may also contribute to heterogeneity within the class. For example, quinazoline derivatives, such as terazosin, have been reported to enhance glycolysis via phosphoglycerate kinase-1, increasing cellular ATP and showing neuroprotective signals in preclinical and early clinical contexts, which is an effect that could counterbalance potential detriments from central α1 antagonism [41,42,43]. By contrast, nonselective, BBB-penetrant compounds (e.g., doxazosin, prazosin) may exert broader central actions than α1A-selective agents with low CNS exposure (e.g., tamsulosin) [44]. Finally, BBB transport depends on passive permeability and efflux (e.g., P-glycoprotein), and BBB integrity declines with aging and Alzheimer’s disease [45,46]. Thus, interindividual variation in CNS exposure to the same drug could help explain inconsistent epidemiologic findings.

Beyond receptor selectivity and BBB permeability, recent pharmacodynamic and neuroimaging studies have offered convergent evidence for biologically plausible cognitive effects. In a 2024 pilot dose-finding study of neurologically healthy adults, terazosin increased whole-blood ATP, consistent with phosphoglycerate kinase-1 activation, and was accompanied by a reduction in global cerebral 18F-FDG uptake on PET, supporting central bioenergetic target engagement [47]. Complementarily, PET work with R-11C-verapamil demonstrates that P-glycoprotein-mediated efflux at the BBB declines with aging and is impaired in Alzheimer’s disease, implying greater brain exposure to BBB-permeant α1-blockers in vulnerable populations [48,49]. Neuromelanin-sensitive MRI links locus coeruleus integrity to cognitive performance in aging/early neurodegeneration, underscoring the relevance of central noradrenergic tone for cognitive resilience [50,51]. In parallel, arterial spin-labeling MRI and related literature connect orthostatic hypotension and cerebral hypoperfusion to cognitive deficits, providing a vascular pathway through which peripherally and centrally acting α1-blockers might indirectly influence cognition [52,53]. Notably, direct human α1-adrenoceptor occupancy imaging remains limited due to the lack of robust PET ligands, so these pharmacodynamic and imaging biomarkers currently serve as informative surrogates to contextualize heterogeneous epidemiologic findings [54,55].

This study has several limitations. First, as with all database studies, inherent issues, such as misclassification bias, uncertainty in causal inference, and selection bias, may still be present. All outcomes of this study were identified based on ICD-10 diagnostic codes, which could introduce a potential risk of misclassification or diagnostic inaccuracy. The TriNetX platform does not allow access to individual patient records or chart-level validation to protect patient privacy and comply with data governance policies. Conversely, ICD coding in the TriNetX network is performed by qualified healthcare professionals within participating institutions and is regularly audited for billing and research purposes. These processes help ensure data integrity, although some degree of misclassification bias cannot be completely excluded. Nevertheless, the TriNetX database is continuously updated and has been utilized in numerous high-quality studies [56], supporting its credibility and reliability. Second, the outcomes in this study were identified using diagnostic codes that could carry the risk of misclassification or misdiagnosis. Although a wide range of covariates was adjusted for, residual confounding from unmeasured variables could still influence the results. In particular, medication adherence, duration of drug exposure, and concomitant antihypertensive therapies could not be fully captured, potentially affecting the observed associations. Third, this study focused exclusively on comparisons between tamsulosin and other α-blockers, without conducting pairwise analyses among the remaining agents. This limitation reduces the ability to generalize the findings across different drug classes and may overlook important pharmacological differences between agents. Lastly, this study was unable to directly assess receptor activity in the brain or measure neurotransmitter concentrations, limiting its ability to provide mechanistic evidence. This limitation highlights the need for future animal studies to validate our findings and further elucidate the underlying biological mechanisms.

In this large-scale, real-world cohort study, we found that different α-blockers are associated with varying risks of dementia, depression, and unintentional injuries. Among the evaluated agents, alfuzosin demonstrated the most favorable risk profile, while prazosin and tamsulosin were linked to a higher risk of neuropsychiatric outcomes. These findings underscore the importance of individualized α-blocker selection, particularly for patients with underlying vulnerability to CNS disorders. By minimizing exposure to agents with potentially adverse neuropsychiatric effects, clinicians may improve long-term outcomes for patients requiring α-blocker therapy. Further research—especially mechanistic and prospective studies—is warranted to confirm these associations and to better understand the underlying biological pathways.

## Data Availability

Data used in this analysis are de-identified and publicly available on the TriNetX website accessed on 22 December 2024 (https://trinetx.com/). Data are available to bona fide researchers who request it from the authors.

## Supplementary Materials

The following supporting information can be downloaded at: https://www.mdpi.com/article/10.3390/jcm14238302/s1. Figure S1. Cohort selection criteria for participants without prior α-blocker use. This figure illustrates the inclusion and exclusion processes used to define the final study population. Participants with a history of other α-blocker use were excluded to ensure a clean comparison between the exposure groups. The flowchart details the se-quential filtering steps applied to reach the final analytic cohort; Figure S2. Hazard ratios for dementia, depression, and unintentional injury in cohorts excluding participants with prior α-blocker use. This figure presents the hazard ra-tios and 95% confidence intervals for the three primary outcomes—dementia, depression, and unintentional injury—comparing tamsulosin with other α-blockers in the main analysis cohort. The results are presented across multiple follow-up durations to assess the consistency of the associations over time; Figure S3. Cohort selection criteria for participants with a history of other α-blocker use or malignancy. This figure illustrates the inclusion and exclusion processes for participants in the sensitivity analyses, which included individuals with a prior history of other α-blocker use or a history of malignancy, leading to the final study pop-ulation for each cohort; Figure S4. Hazard ratios for dementia, depression, and unintentional injury in cohorts including participants with a history of other α-blocker use or malignancy. This fig-ure presents the hazard ratios and 95% confidence intervals for the primary outcomes—dementia, depression, and unintentional injury—comparing tamsulosin with other α-blockers in the sensitivity analyses, which included participants with a prior history of other α-blocker use or malignancy; Table S1. Definition of Inclusion and Exclusion Criteria and Outcomes; Table S2. Demographic and clinical characteristics before and after matching between Cohort A and Cohort B; Table S3. Demographic and clinical characteristics before and after matching between Cohort A and Cohort C; Table S4. Demographic and clinical characteristics before and after matching between Cohort A and Cohort D; Table S5. Demographic and clinical characteristics before and after matching between Cohort A and Cohort E; Table S6. Demographic and clinical characteristics before and after matching between Cohort A and Cohort F; Table S7. Demographic and clinical characteristics before and after matching between Cohort G and Cohort H; Table S8. Demographic and clinical characteristics before and after matching between Cohort G and Cohort I; Table S9. Demographic and clinical characteristics before and after matching between Cohort G and Cohort J; Table S10. Demographic and clinical characteristics before and after matching between Cohort G and Cohort K; Table S11. Demographic and clinical characteristics before and after matching between Cohort G and Cohort L; Table S12. Demographic and clinical characteristics before and after matching between Cohort M and Cohort N; Table S13. Demographic and clinical characteristics before and after matching between Cohort M and Cohort O; Table S14. Demographic and clinical characteristics before and after matching between Cohort M and Cohort P; Table S15. Demographic and clinical characteristics before and after matching between Cohort M and Cohort Q; Table S16. Demographic and clinical characteristics before and after matching between Cohort M and Cohort R.

## References

[1] Stafford R.S., Furberg C.D., Finkelstein S.N., Cockburn I.M., Alehegn T., Ma J. (2004). Impact of clinical trial results on national trends in alpha-blocker prescribing, 1996–2002. JAMA.

[2] McVary K.T., Roehrborn C.G., Avins A.L., Barry M.J., Bruskewitz R.C., Donnell R.F., Foster H.E., Gonzalez C.M., Kaplan S.A., Penson D.F. (2011). Update on AUA guideline on the management of benign prostatic hyperplasia. J. Urol..

[3] Hollingsworth J.M., Wilt T.J. (2014). Lower urinary tract symptoms in men. BMJ.

[4] Filson C.P., Hollingsworth J.M., Clemens J.Q., Wei J.T. (2013). The efficacy and safety of combined therapy with α-blockers and anticholinergics for men with benign prostatic hyperplasia: A meta-analysis. J. Urol..

[5] Oelke M., Becher K., Castro-Diaz D., Chartier-Kastler E., Kirby M., Wagg A., Wehling M. (2015). Appropriateness of oral drugs for long-term treatment of lower urinary tract symptoms in older persons: Results of a systematic literature review and international consensus validation process (LUTS-FORTA 2014). Age Ageing.

[6] Aurora R.N., Zak R.S., Auerbach S.H., Casey K.R., Chowdhuri S., Karippot A., Maganti R.K., Ramar K., Kristo D.A., Bista S.R. (2010). Best practice guide for the treatment of nightmare disorder in adults. J. Clin. Sleep. Med..

[7] Raskind M.A., Peskind E.R., Chow B., Harris C., Davis-Karim A., Holmes H.A., Hart K.L., McFall M., Mellman T.A., Reist C. (2018). Trial of Prazosin for Post-Traumatic Stress Disorder in Military Veterans. N. Engl. J. Med..

[8] Muderrisoglu A.E., Becher K.F., Madersbacher S., Michel M.C. (2019). Cognitive and mood side effects of lower urinary tract medication. Expert. Opin. Drug Saf..

[9] Duan Y., Grady J.J., Albertsen P.C., Helen Wu Z. (2018). Tamsulosin and the risk of dementia in older men with benign prostatic hyperplasia. Pharmacoepidemiol. Drug Saf..

[10] Yeon B., Suh A.Y., Choi E., Kim B., Noh E., Chung S.Y., Han S.Y. (2022). Depression risk associated with the use of 5α-reductase inhibitors versus α-blockers: A retrospective cohort study in South Korea. PLoS ONE.

[11] Beers M.H., Passman L.J. (1990). Antihypertensive medications and depression. Drugs.

[12] Welk B., McArthur E., Fraser L.A., Hayward J., Dixon S., Hwang Y.J., Ordon M. (2015). The risk of fall and fracture with the initiation of a prostate-selective α antagonist: A population based cohort study. BMJ.

[13] Souverein P.C., Van Staa T.P., Egberts A.C., De la Rosette J.J., Cooper C., Leufkens H.G. (2003). Use of alpha-blockers and the risk of hip/femur fractures. J. Intern. Med..

[14] Hart A., Aldridge G., Zhang Q., Narayanan N.S., Simmering J.E. (2024). Association of Terazosin, Doxazosin, or Alfuzosin Use and Risk of Dementia With Lewy Bodies in Men. Neurology.

[15] Sohn J.H., Lee S.H., Kwon Y.S., Kim J.H., Kim Y., Lee J.J. (2020). The impact of tamsulosin on cognition in Alzheimer disease with benign prostate hyperplasia: A study using the Hallym Smart Clinical Data Warehouse. Medicine.

[16] Fung K.W., Baye F., Baik S.H., McDonald C.J. (2024). Tamsulosin use in benign prostatic hyperplasia and risks of Parkinson’s disease, Alzheimer’s disease and mortality: An observational cohort study of elderly Medicare enrollees. PLoS ONE.

[17] Tae B.S., Jeon B.J., Choi H., Cheon J., Park J.Y., Bae J.H. (2019). α-Blocker and Risk of Dementia in Patients with Benign Prostatic Hyperplasia: A Nationwide Population Based Study Using the National Health Insurance Service Database. J. Urol..

[18] Latvala L., Tiihonen M., Murtola T.J., Hartikainen S., Tolppanen A.M. (2022). Use of α1-adrenoceptor antagonists tamsulosin and alfuzosin and the risk of Alzheimer’s disease. Pharmacoepidemiol. Drug Saf..

[19] Garcia-Argibay M., Hiyoshi A., Fall K., Montgomery S. (2022). Association of 5α-Reductase Inhibitors With Dementia, Depression, and Suicide. JAMA Netw. Open.

[20] Sommerlad A., Ruegger J., Singh-Manoux A., Lewis G., Livingston G. (2018). Marriage and risk of dementia: Systematic review and meta-analysis of observational studies. J. Neurol. Neurosurg. Psychiatry.

[21] Hendriks S., Ranson J.M., Peetoom K., Lourida I., Tai X.Y., de Vugt M., Llewellyn D.J., Köhler S. (2024). Risk Factors for Young-Onset Dementia in the UK Biobank. JAMA Neurol..

[22] Doze V.A., Papay R.S., Goldenstein B.L., Gupta M.K., Collette K.M., Nelson B.W., Lyons M.J., Davis B.A., Luger E.J., Wood S.G. (2011). Long-term α1A-adrenergic receptor stimulation improves synaptic plasticity, cognitive function, mood, and longevity. Mol. Pharmacol..

[23] Doze V.A., Handel E.M., Jensen K.A., Darsie B., Luger E.J., Haselton J.R., Talbot J.N., Rorabaugh B.R. (2009). alpha(1A)- and alpha(1B)-adrenergic receptors differentially modulate antidepressant-like behavior in the mouse. Brain Res..

[24] Koh W., Park M., Chun Y.E., Lee J., Shim H.S., Park M.G., Kim S., Sa M., Joo J., Kang H. (2022). Astrocytes Render Memory Flexible by Releasing D-Serine and Regulating NMDA Receptor Tone in the Hippocampus. Biol. Psychiatry.

[25] Franco-Salinas G., de la Rosette J.J., Michel M.C. (2010). Pharmacokinetics and pharmacodynamics of tamsulosin in its modified-release and oral controlled absorption system formulations. Clin. Pharmacokinet..

[26] Nikolic K., Filipic S., Smoliński A., Kaliszan R., Agbaba D. (2013). Partial least square and hierarchical clustering in ADMET modeling: Prediction of blood-brain barrier permeation of α-adrenergic and imidazoline receptor ligands. J. Pharm. Pharm. Sci..

[27] Valletta M., Vetrano D.L., Xia X., Rizzuto D., Roso-Llorach A., Calderón-Larrañaga A., Marengoni A., Laukka E.J., Canevelli M., Bruno G. (2023). Multimorbidity patterns and 18-year transitions from normal cognition to dementia and death: A population-based study. J. Intern. Med..

[28] Mo M., Zacarias-Pons L., Hoang M.T., Mostafaei S., Jurado P.G., Stark I., Johnell K., Eriksdotter M., Xu H., Garcia-Ptacek S. (2023). Psychiatric Disorders Before and After Dementia Diagnosis. JAMA Netw. Open.

[29] Perez D.M. (2020). α(1)-Adrenergic Receptors in Neurotransmission, Synaptic Plasticity, and Cognition. Front. Pharmacol..

[30] Dewa K.I., Kaseda K., Kuwahara A., Kubotera H., Yamasaki A., Awata N., Komori A., Holtz M.A., Kasai A., Skibbe H. (2025). The Astrocytic Ensemble Acts as a Multiday Trace to Stabilize Memory. Nature.

[31] Perez D.M. (2021). Current Developments on the Role of α(1)-Adrenergic Receptors in Cognition, Cardioprotection, and Metabolism. Front. Cell Dev. Biol..

[32] Wahis J., Holt M.G. (2021). Astrocytes, Noradrenaline, α1-Adrenoreceptors, and Neuromodulation: Evidence and Unanswered Questions. Front. Cell. Neurosci..

[33] Purkayastha S., Raven P.B. (2011). The functional role of the alpha-1 adrenergic receptors in cerebral blood flow regulation. Indian J. Pharmacol..

[34] Ma Y., Zhang Y., Coresh J., Viswanathan A., Sullivan K.J., Walker K.A., Liu C., Lipsitz L.A., Selvin E., Sharrett A.R. (2024). Orthostatic Blood Pressure Change, Dizziness, and Risk of Dementia in the ARIC Study. Hypertension.

[35] Sara S.J. (2009). The locus coeruleus and noradrenergic modulation of cognition. Nat. Rev. Neurosci..

[36] Berridge C.W., Schmeichel B.E., España R.A. (2012). Noradrenergic modulation of wakefulness/arousal. Sleep. Med. Rev..

[37] Pudovkina O.L., Westerink B.H. (2005). Functional role of alpha1-adrenoceptors in the locus coeruleus: A microdialysis study. Brain Res..

[38] Collins L., Francis J., Emanuel B., McCormick D.A. (2023). Cholinergic and noradrenergic axonal activity contains a behavioral-state signal that is coordinated across the dorsal cortex. eLife.

[39] Heneka M.T., Nadrigny F., Regen T., Martinez-Hernandez A., Dumitrescu-Ozimek L., Terwel D., Jardanhazi-Kurutz D., Walter J., Kirchhoff F., Hanisch U.K. (2010). Locus ceruleus controls Alzheimer’s disease pathology by modulating microglial functions through norepinephrine. Proc. Natl. Acad. Sci. USA.

[40] Zou H.L., Li J., Zhou J.L., Yi X., Cao S. (2021). Effects of norepinephrine on microglial neuroinflammation and neuropathic pain. Ibrain.

[41] Cai R., Zhang Y., Simmering J.E., Schultz J.L., Li Y., Fernandez-Carasa I., Consiglio A., Raya A., Polgreen P.M., Narayanan N.S. (2019). Enhancing glycolysis attenuates Parkinson’s disease progression in models and clinical databases. J. Clin. Investig.

[42] Schultz J.L., Brinker A.N., Xu J., Ernst S.E., Tayyari F., Rauckhorst A.J., Liu L., Uc E.Y., Taylor E.B., Simmering J.E. (2022). A pilot to assess target engagement of terazosin in Parkinson’s disease. Park. Relat. Disord..

[43] Weber M.A., Sivakumar K., Tabakovic E.E., Oya M., Aldridge G.M., Zhang Q., Simmering J.E., Narayanan N.S. (2023). Glycolysis-enhancing α(1)-adrenergic antagonists modify cognitive symptoms related to Parkinson’s disease. npj Park. Dis..

[44] Richardson C.D., Donatucci C.F., Page S.O., Wilson K.H., Schwinn D.A. (1997). Pharmacology of tamsulosin: Saturation-binding isotherms and competition analysis using cloned alpha 1-adrenergic receptor subtypes. Prostate.

[45] Kim Y.J., Tae B.S., Bae J.H. (2020). Cognitive Function and Urologic Medications for Lower Urinary Tract Symptoms. Int. Neurourol. J..

[46] Hudson S.M., Whiteside T.E., Lorenz R.A., Wargo K.A. (2012). Prazosin for the treatment of nightmares related to posttraumatic stress disorder: A review of the literature. Prim. Care Companion CNS Disord..

[47] Schultz J.L., Gander P.E., Workman C.D., Ponto L.L., Cross S., Nance C.S., Groth C.L., Taylor E.B., Ernst S.E., Xu J. (2024). A pilot dose-finding study of Terazosin in humans. medRxiv.

[48] van Assema D.M.E., Lubberink M., Bauer M., van der Flier W.M., Schuit R.C., Windhorst A.D., Comans E.F.I., Hoetjes N.J., Tolboom N., Langer O. (2011). Blood–brain barrier P-glycoprotein function in Alzheimer’s disease. Brain.

[49] van Assema D.M., Lubberink M., Boellaard R., Schuit R.C., Windhorst A.D., Scheltens P., Lammertsma A.A., van Berckel B.N. (2012). P-glycoprotein function at the blood-brain barrier: Effects of age and gender. Mol. Imaging Biol..

[50] Bennett I.J., Langley J., Sun A., Solis K., Seitz A.R., Hu X.P. (2024). Locus coeruleus contrast and diffusivity metrics differentially relate to age and memory performance. Sci. Rep..

[51] Trujillo P., Aumann M.A., Claassen D.O. (2024). Neuromelanin-sensitive MRI as a promising biomarker of catecholamine function. Brain.

[52] Ferreira R., Bastos-Leite A.J. (2024). Arterial spin labelling magnetic resonance imaging and perfusion patterns in neurocognitive and other mental disorders: A systematic review. Neuroradiology.

[53] Gottesman R.F., Egle M., Groechel R.C., Mughal A. (2025). Blood pressure and the brain: The conundrum of hypertension and dementia. Cardiovasc. Res..

[54] Alluri S.R., Kim S.W., Volkow N.D., Kil K.E. (2020). PET Radiotracers for CNS-Adrenergic Receptors: Developments and Perspectives. Molecules.

[55] Risgaard R., Ettrup A., Balle T., Dyssegaard A., Hansen H.D., Lehel S., Madsen J., Pedersen H., Püschl A., Badolo L. (2013). Radiolabelling and PET brain imaging of the α_1_-adrenoceptor antagonist Lu AE43936. Nucl. Med. Biol..

[56] Taquet M., Luciano S., Geddes J.R., Harrison P.J. (2021). Bidirectional associations between COVID-19 and psychiatric disorder: Retrospective cohort studies of 62 354 COVID-19 cases in the USA. Lancet Psychiatry.

