# Supplementary material for: Differential Risks of Dementia, Depression, and Injury Among Common α-Blockers, with Tamsulosin as the Reference Drug: A Real-World Cohort Study in Men with Lower Urinary Tract Symptoms
Source: J Clin Med. 2025 Nov 22;14(23):8302. doi: 10.3390/jcm14238302 (PMC12693173; doi:10.3390/jcm14238302)
Supplement: Supplementary file 1 [file jcm-14-08302-s001.zip › jcm-3921094-supplementary.pdf]

Journal of
Clinical Medicine

MDPI

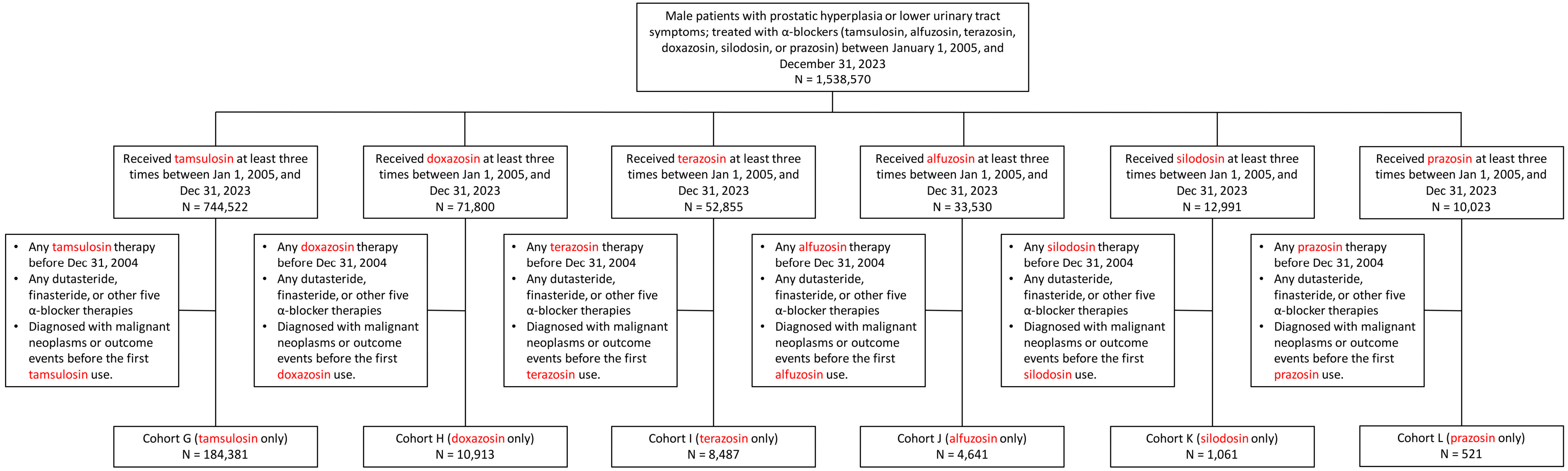

Figure S1. Cohort selection criteria for participants without prior α-blocker use. This figure illustrates the inclusion and exclusion processes used to define the final study population. Participants with a history of other α-blocker use were excluded to ensure a clean comparison between the exposure groups. The flowchart details the sequential filtering steps applied to reach the final analytic cohort.

(A)

Disease	Patients with outcome (Cohort G /Cohort H)	Hazard Ratio (95% CI)[2] Cohort A as reference	Patients with outcome (Cohort G /Cohort I)	Hazard Ratio (95% CI)[2] Cohort A as reference
**F01-F03 Dementia**				
1y	191/115	0.595 (0.472-0.750)	174/103	0.584 (0.458-0.745)
3y	392/264	0.659 (0.564-0.770)	370/239	0.630 (0.535-0.741)
10y	757/584	0.748 (0.671-0.833)	695/505	0.701 (0.625-0.786)
**F01-F09 Mental disorders due to known physiological conditions**				
1y	254/180	0.700 (0.579-0.848)	243/147	0.596 (0.486-0.732)
3y	536/390	0.712 (0.625-0.811)	477/327	0.667 (0.580-0.768)
10y	994/807	0.785 (0.716-0.862)	887/665	0.722 (0.653-0.799)
**F30-F39 Mood [affective] disorders**				
1y	487/402	0.815 (0.714-0.930)	381/332	0.861 (0.743-0.997)
3y	940/815	0.849 (0.773-0.933)	717/653	0.890 (0.800-0.989)
10y	1498/1340	0.872 (0.810-0.939)	1190/1081	0.881 (0.811-0.956)
**F32-F33 Depressive**				
1y	438/362	0.816 (0.710-0.937)	337/297	0.871 (0.745-1.018)
3y	855/740	0.849 (0.769-0.936)	652/601	0.901 (0.807-1.007)
10y	1381/1235	0.873 (0.808-0.943)	1102/1010	0.889 (0.817-0.969)
**F40-F48 Anxiety-dissociative-stress-related-somatoform and other nonpsychotic mental disorders**				
1y	472/427	0.895 (0.786-1.021)	365/307	0.830 (0.713-0.966)
3y	925/886	0.942 (0.859-1.033)	731/645	0.859 (0.772-0.955)
10y	1614/1498	0.906 (0.845-0.972)	1274/1154	0.872 (0.805-0.944)
**F41 Other anxiety disorders**				
1y	392/345	0.871 (0.754-1.007)	298/241	0.798 (0.674-0.946)
3y	744/696	0.920 (0.829-1.020)	585/497	0.828 (0.734-0.933)
10y	1257/1173	0.912 (0.842-0.987)	991/868	0.843 (0.770-0.924)
**G30 Alzheimer's disease**				
1y	48/25	0.516 (0.318-0.836)	45/29	0.638 (0.400-1.018)
3y	109/65	0.585 (0.430-0.795)	99/71	0.702 (0.518-0.953)
10y	233/161	0.673 (0.551-0.823)	211/156	0.717 (0.583-0.882)
**G30-G32 Other degenerative diseases of the nervous system**				
1y	133/85	0.632 (0.481-0.829)	127/80	0.622 (0.471-0.823)
3y	302/214	0.694 (0.582-0.827)	278/195	0.684 (0.570-0.822)
10y	647/502	0.753 (0.670-0.846)	601/413	0.661 (0.584-0.750)
**S00-S09 Injuries to the head**				
1y	217/189	0.862 (0.709-1.048)	186/169	0.899 (0.730-1.107)
3y	544/472	0.850 (0.752-0.962)	446/393	0.861 (0.752-0.986)
10y	1163/1015	0.845 (0.777-0.919)	998/833	0.806 (0.735-0.883)
**S70-S79 Injuries to the hip and thigh**				
1y	119/87	0.723 (0.549-0.954)	90/75	0.824 (0.607-1.120)
3y	276/247	0.878 (0.740-1.043)	231/183	0.775 (0.639-0.941)
10y	576/545	0.922 (0.820-1.037)	510/414	0.785 (0.689-0.893)

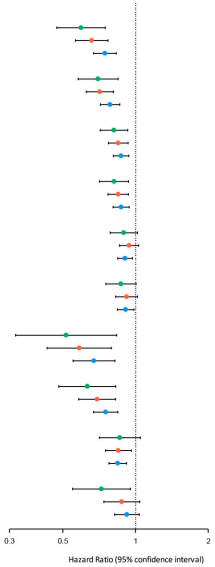

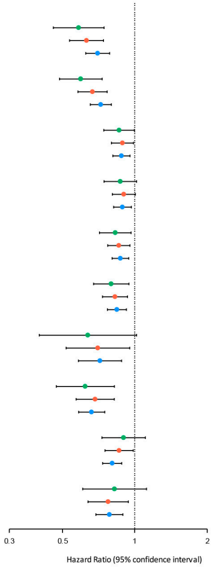

(B)

Disease	Patients with outcome (Cohort G /Cohort J)	Hazard Ratio (95% CI)[2] Cohort A as reference	Patients with outcome (Cohort G /Cohort K)	Hazard Ratio (95% CI)[2] Cohort A as reference	Patients with outcome (Cohort G /Cohort L)	Hazard Ratio (95% CI)[2] Cohort A as reference
**F01-F03 Dementia**						
1y	46/18	0.379 (0.220-0.653)	11/≤10	0.628 (0.243-1.620)	≤10/≤10	1.004 (0.324-3.112)
3y	100/47	0.451 (0.319-0.638)	25/16	0.635 (0.339-1.189)	≤10/15	2.161 (0.881-5.300)
10y	171/83	0.471 (0.362-0.612)	41/30	0.732 (0.457-1.172)	17/20	1.184 (0.620-2.260)
**F01-F09 Mental disorders due to known physiological conditions**						
1y	65/36	0.537 (0.357-0.806)	19/12	0.622 (0.302-1.281)	≤10/≤10	0.801 (0.316-2.029)
3y	141/70	0.477 (0.358-0.635)	37/21	0.562 (0.329-0.959)	14/21	1.509 (0.768-2.968)
10y	239/118	0.478 (0.383-0.596)	59/47	0.800 (0.545-1.174)	27/34	1.276 (0.770-2.114)
**F30-F39 Mood [affective] disorders**						
1y	231/117	0.484 (0.388-0.605)	48/33	0.672 (0.431-1.047)	27/78	3.032 (1.957-4.696)
3y	419/234	0.528 (0.450-0.620)	103/55	0.523 (0.377-0.725)	50/142	3.161 (2.289-4.363)
10y	616/357	0.548 (0.481-0.624)	139/78	0.548 (0.415-0.723)	73/182	2.889 (2.202-3.792)
**F32-F33 Depressive**						
1y	210/107	0.488 (0.387-0.616)	41/30	0.717 (0.448-1.148)	25/63	2.619 (1.648-4.163)
3y	378/214	0.536 (0.454-0.634)	92/54	0.577 (0.413-0.808)	48/124	2.823 (2.023-3.940)
10y	566/328	0.550 (0.480-0.630)	129/74	0.563 (0.423-0.749)	70/161	2.602 (1.965-3.445)
**F40-F48 Anxiety-dissociative-stress-related-somatoform and other nonpsychotic mental disorders**						
1y	243/136	0.536 (0.435-0.661)	49/34	0.677 (0.437-1.049)	28/80	3.038 (1.975-4.671)
3y	463/292	0.596 (0.515-0.691)	99/68	0.676 (0.497-0.921)	51/135	2.922 (2.117-4.033)
10y	708/468	0.624 (0.556-0.702)	155/116	0.736 (0.578-0.936)	86/178	2.331 (1.802-3.015)
**F41 Other anxiety disorders**						
1y	205/107	0.501 (0.396-0.632)	41/28	0.667 (0.413-1.079)	25/47	1.941 (1.195-3.153)
3y	388/230	0.562 (0.477-0.661)	83/54	0.640 (0.454-0.901)	42/85	2.120 (1.465-3.068)
10y	568/363	0.607 (0.532-0.693)	127/103	0.804 (0.620-1.043)	75/124	1.762 (1.323-2.347)
**G30 Alzheimer's disease**						
1y	≤10/≤10	2.277 (0.589-8.806)	≤10/≤10	2.963 (0.308-28.489)	0/0	N/A
3y	12/16	1.289 (0.610-2.725)	≤10/≤10	1.745 (0.511-5.961)	0/0	N/A
10y	36/33	0.902 (0.562-1.446)	11/15	1.379 (0.633-3.003)	≤10/≤10	3.237 (0.336-31.178)
**G30-G32 Other degenerative diseases of the nervous system**						
1y	36/16	0.430 (0.239-0.775)	≤10/≤10	1.110 (0.428-2.876)	≤10/≤10	4.028 (0.450-36.035)
3y	77/43	0.537 (0.369-0.779)	21/19	0.901 (0.484-1.676)	≤10/13	13.172 (1.723-100.694)
10y	166/93	0.546 (0.424-0.704)	40/33	0.830 (0.524-1.316)	≤10/20	2.054 (0.961-4.389)
**S00-S09 Injuries to the head**						
1y	82/40	0.470 (0.322-0.686)	20/11	0.538 (0.258-1.123)	≤10/19	2.762 (1.161-6.571)
3y	170/125	0.704 (0.559-0.886)	44/24	0.538 (0.327-0.885)	18/41	2.351 (1.351-4.092)
10y	352/227	0.623 (0.527-0.736)	83/43	0.514 (0.355-0.742)	40/88	2.357 (1.621-3.425)
**S70-S79 Injuries to the hip and thigh**						
1y	42/30	0.690 (0.432-1.103)	≤10/≤10	0.883 (0.359-2.174)	≤10/11	2.212 (0.769-6.368)
3y	102/76	0.717 (0.533-0.965)	25/14	0.554 (0.288-1.065)	13/20	1.551 (0.772-3.118)
10y	191/132	0.674 (0.540-0.841)	50/34	0.677 (0.438-1.046)	29/37	1.316 (0.810-2.141)

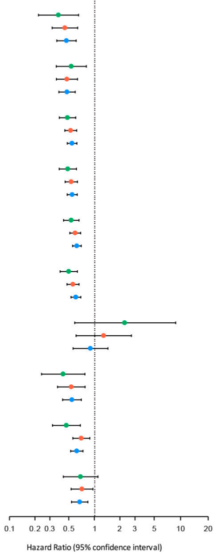

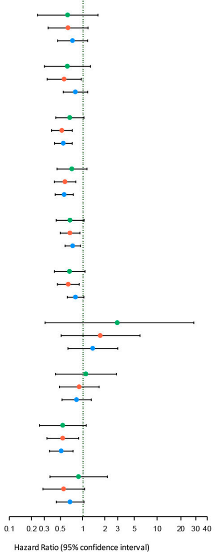

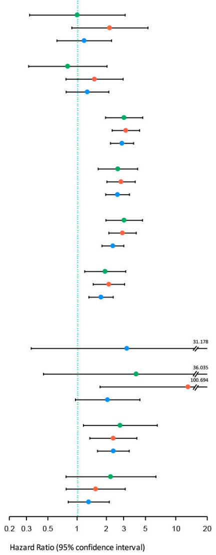

Figure S2. Hazard ratios for dementia, depression, and unintentional injury in cohorts excluding participants with prior α-blocker use. This figure presents the hazard ratios and 95% confidence intervals for the three primary outcomes—dementia, depression, and unintentional injury—comparing tamsulosin with other α-blockers in the main analysis cohort. The results are presented across multiple follow-up durations to assess the consistency of the associations over time.

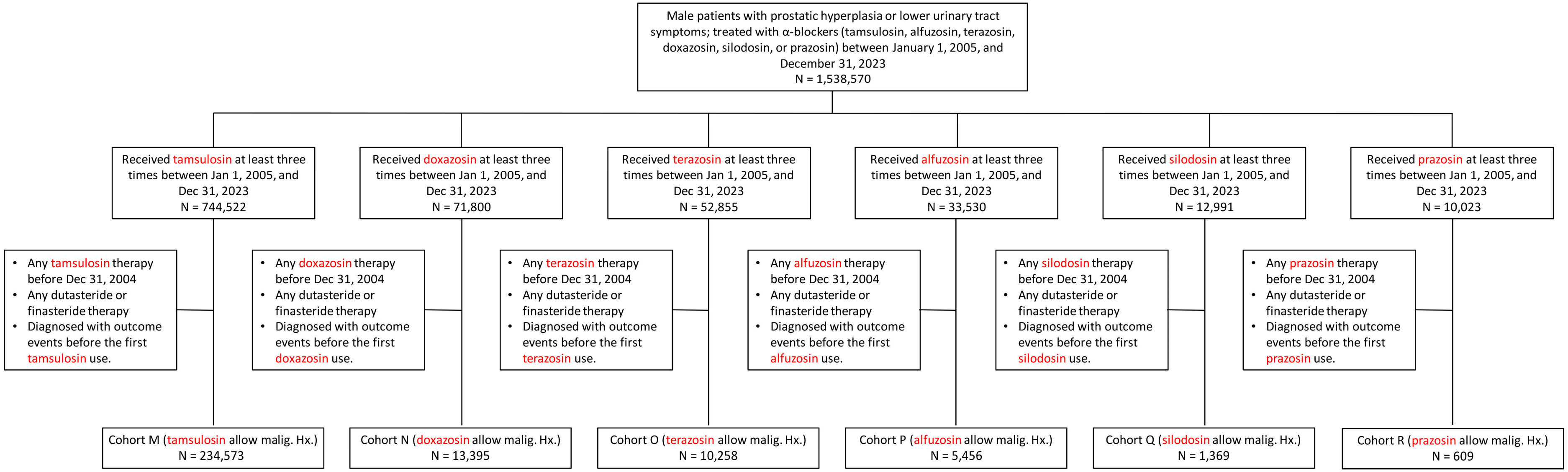

Figure S3. Cohort selection criteria for participants with a history of other α-blocker use or malignancy. This figure illustrates the inclusion and exclusion processes for participants in the sensitivity analyses, which included individuals with a prior history of other α-blocker use or a history of malignancy, leading to the final study population for each cohort.

(A)

Disease	Patients with outcome (Cohort M /Cohort N)	Hazard Ratio (95% CI)[2] Cohort A as reference	Patients with outcome (Cohort M /Cohort O)	Hazard Ratio (95% CI)[2] Cohort A as reference
**F01-F03 Dementia**				
1y	223/138	0.612 (0.495-0.757)	193/125	0.643 (0.513-0.805)
3y	480/318	0.650 (0.564-0.749)	409/294	0.708 (0.609-0.822)
10y	913/701	0.748 (0.678-0.826)	778/619	0.769 (0.692-0.855)
**F01-F09 Mental disorders due to known physiological conditions**				
1y	324/217	0.662 (0.557-0.786)	269/193	0.712 (0.592-0.857)
3y	675/483	0.701 (0.624-0.788)	565/417	0.726 (0.640-0.824)
10y	1237/982	0.772 (0.710-0.839)	1043/821	0.760 (0.694-0.833)
**F30-F39 Mood [affective] disorders**				
1y	598/476	0.786 (0.697-0.886)	477/406	0.845 (0.740-0.964)
3y	1138/979	0.844 (0.775-0.919)	898/783	0.858 (0.780-0.945)
10y	1807/1607	0.869 (0.812-0.929)	1433/1287	0.874 (0.810-0.942)
**F32-F33 Depressive**				
1y	544/424	0.769 (0.678-0.873)	438/359	0.813 (0.707-0.935)
3y	1049/886	0.829 (0.758-0.906)	826/717	0.854 (0.773-0.944)
10y	1673/1474	0.861 (0.803-0.923)	1322/1198	0.882 (0.816-0.954)
**F40-F48 Anxiety-dissociative-stress-related-somatoform and other nonpsychotic mental disorders**				
1y	531/511	0.954 (0.845-1.078)	426/391	0.911 (0.794-1.045)
3y	1094/1083	0.978 (0.899-1.064)	861/792	0.906 (0.823-0.998)
10y	1936/1837	0.933 (0.875-0.994)	1456/1389	0.925 (0.860-0.996)
**F41 Other anxiety disorders**				
1y	419/403	0.954 (0.832-1.094)	339/305	0.894 (0.766-1.044)
3y	855/840	0.970 (0.882-1.067)	685/605	0.871 (0.780-0.971)
10y	1471/1424	0.952 (0.885-1.024)	1134/1037	0.887 (0.815-0.965)
**G30 Alzheimer's disease**				
1y	42/28	0.661 (0.410-1.066)	53/37	0.695 (0.456-1.057)
3y	120/75	0.615 (0.461-0.820)	103/92	0.883 (0.666-1.169)
10y	267/193	0.707 (0.588-0.851)	234/200	0.830 (0.688-1.003)
**G30-G32 Other degenerative diseases of the nervous system**				
1y	139/105	0.748 (0.580-0.964)	146/103	0.701 (0.545-0.902)
3y	370/265	0.702 (0.600-0.822)	312/251	0.792 (0.671-0.936)
10y	802/624	0.757 (0.681-0.840)	673/522	0.749 (0.668-0.840)
**S00-S09 Injuries to the head**				
1y	275/237	0.853 (0.717-1.015)	223/208	0.929 (0.769-1.122)
3y	655/595	0.892 (0.799-0.997)	546/498	0.898 (0.795-1.014)
10y	1427/1254	0.855 (0.792-0.922)	1172/1055	0.868 (0.799-0.944)
**S70-S79 Injuries to the hip and thigh**				
1y	141/112	0.787 (0.614-1.009)	112/99	0.880 (0.671-1.153)
3y	352/305	0.853 (0.731-0.994)	281/238	0.837 (0.704-0.995)
10y	701/682	0.953 (0.857-1.059)	560/515	0.893 (0.793-1.007)

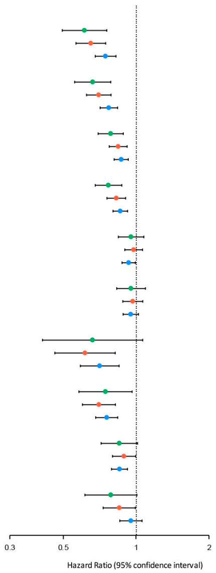

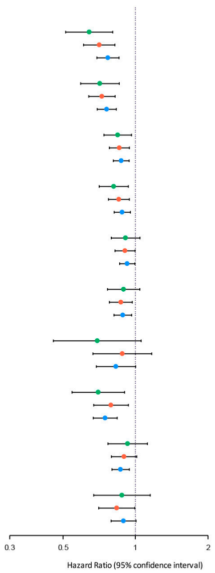

(B)

Disease	Patients with outcome (Cohort M /Cohort P)	Hazard Ratio (95% CI)[2] Cohort A as reference	Patients with outcome (Cohort M /Cohort Q)	Hazard Ratio (95% CI)[2] Cohort A as reference	Patients with outcome (Cohort M /Cohort R)	Hazard Ratio (95% CI)[2] Cohort A as reference
**F01-F03 Dementia**						
1y	62/24	0.373 (0.233-0.597)	12/≤10	0.821 (0.355-1.900)	≤10/≤10	1.006 (0.325-3.120)
3y	126/59	0.448 (0.329-0.611)	32/23	0.704 (0.412-1.204)	12/19	1.601 (0.777-3.298)
10y	236/105	0.430 (0.342-0.541)	55/42	0.748 (0.501-1.118)	19/26	1.406 (0.778-2.542)
**F01-F09 Mental disorders due to known physiological conditions**						
1y	107/47	0.423 (0.300-0.597)	22/18	0.806 (0.432-1.502)	11/11	1.004 (0.435-2.316)
3y	206/92	0.427 (0.334-0.546)	54/31	0.562 (0.361-0.874)	25/30	1.208 (0.711-2.055)
10y	356/147	0.397 (0.328-0.481)	85/67	0.769 (0.558-1.059)	43/46	1.097 (0.724-1.663)
**F30-F39 Mood [affective] disorders**						
1y	239/138	0.551 (0.447-0.680)	68/42	0.603 (0.410-0.885)	31/86	2.934 (1.946-4.423)
3y	458/266	0.549 (0.472-0.639)	129/75	0.563 (0.424-0.748)	62/157	2.822 (2.103-3.787)
10y	701/411	0.552 (0.489-0.623)	179/103	0.551 (0.433-0.702)	84/207	2.901 (2.250-3.739)
**F32-F33 Depressive**						
1y	208/127	0.584 (0.469-0.729)	62/38	0.599 (0.400-0.897)	29/70	2.523 (1.636-3.889)
3y	414/244	0.559 (0.477-0.654)	118/73	0.600 (0.448-0.804)	59/139	2.579 (1.902-3.497)
10y	636/380	0.565 (0.498-0.642)	165/97	0.565 (0.440-0.726)	81/186	2.647 (2.038-3.437)
**F40-F48 Anxiety-dissociative-stress-related-somatoform and other nonpsychotic mental disorders**						
1y	261/157	0.575 (0.472-0.701)	62/47	0.745 (0.510-1.088)	38/90	2.512 (1.719-3.671)
3y	529/333	0.595 (0.519-0.683)	124/89	0.699 (0.533-0.918)	77/149	2.115 (1.606-2.785)
10y	799/533	0.632 (0.566-0.705)	192/145	0.726 (0.585-0.901)	107/204	2.150 (1.701-2.717)
**F41 Other anxiety disorders**						
1y	232/125	0.515 (0.415-0.641)	52/39	0.737 (0.487-1.116)	32/54	1.747 (1.128-2.705)
3y	452/261	0.547 (0.469-0.637)	105/72	0.669 (0.495-0.902)	62/96	1.621 (1.178-2.231)
10y	670/411	0.582 (0.514-0.658)	158/128	0.783 (0.620-0.988)	86/142	1.767 (1.352-2.310)
**G30 Alzheimer's disease**						
1y	11/≤10	0.701 (0.282-1.743)	≤10/≤10	3.945 (0.441-35.291)	≤10/0	N/A
3y	27/18	0.639 (0.352-1.161)	≤10/≤10	1.407 (0.536-3.697)	≤10/≤10	0.671 (0.112-4.017)
10y	65/37	0.554 (0.370-0.829)	18/22	1.210 (0.649-2.255)	≤10/≤10	1.260 (0.384-4.132)
**G30-G32 Other degenerative diseases of the nervous system**						
1y	38/22	0.557 (0.329-0.941)	≤10/≤10	0.988 (0.411-2.374)	≤10/≤10	0.844 (0.258-2.767)
3y	93/56	0.575 (0.413-0.802)	22/26	1.165 (0.660-2.056)	≤10/19	1.932 (0.898-4.155)
10y	209/115	0.532 (0.424-0.669)	49/46	0.928 (0.620-1.388)	21/28	1.380 (0.783-2.430)
**S00-S09 Injuries to the head**						
1y	98/50	0.491 (0.349-0.690)	26/16	0.603 (0.324-1.124)	16/23	1.453 (0.768-2.750)
3y	225/150	0.636 (0.518-0.782)	53/35	0.646 (0.422-0.990)	43/51	1.209 (0.806-1.815)
10y	456/269	0.566 (0.487-0.658)	115/63	0.528 (0.388-0.718)	75/105	1.477 (1.098-1.987)
**S70-S79 Injuries to the hip and thigh**						
1y	53/39	0.709 (0.469-1.072)	11/11	0.984 (0.427-2.270)	13/12	0.930 (0.424-2.037)
3y	123/98	0.764 (0.586-0.996)	26/18	0.680 (0.373-1.240)	21/22	1.057 (0.581-1.921)
10y	232/165	0.688 (0.564-0.841)	53/43	0.793 (0.530-1.185)	34/39	1.203 (0.760-1.906)

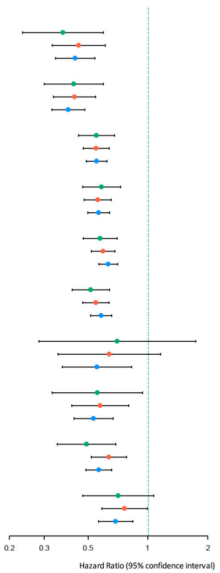

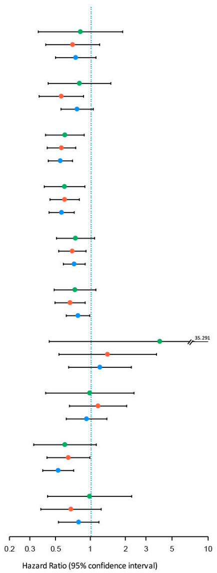

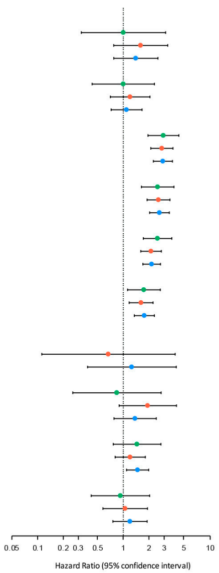

Figure S4. Hazard ratios for dementia, depression, and unintentional injury in cohorts including participants with a history of other α-blocker use or malignancy. This figure presents the hazard ratios and 95% confidence intervals for the primary outcomes—dementia, depression, and unintentional injury—comparing tamsulosin with other α-blockers in the sensitivity analyses, which included participants with a prior history of other α-blocker use or malignancy.

Journal of
Clinical Medicine

Table S1. Definition of Inclusion and Exclusion Criteria and Outcomes.

Definition for “prostatic hyperplasia or lower urinary tract symptoms”	
UMLS:ICD10CM:N40	Benign prostatic hyperplasia
UMLS:ICD10CM:R33	Retention of urine
UMLS:ICD10CM:R35.0	Frequency of micturition
UMLS:ICD10CM:R35.1	Nocturia
Definition for medication	
NLM:RXNORM:17300	alfuzosin
NLM:RXNORM:37798	terazosin
NLM:RXNORM:49276	doxazosin
NLM:RXNORM:720825	silodosin
NLM:RXNORM:77492	tamsulosin
NLM:RXNORM:8629	prazosin
NLM:RXNORM:228790	dutasteride
NLM:RXNORM:25025	finasteride
Definition for malignant neoplasms	
UMLS:ICD10CM:C45-C49	Malignant neoplasms of mesothelial and soft tissue
UMLS:ICD10CM:C50-C50	Malignant neoplasms of breast (C50)
UMLS:ICD10CM:C00-C14	Malignant neoplasms of lip, oral cavity and pharynx
UMLS:ICD10CM:C30-C39	Malignant neoplasms of respiratory and intrathoracic organs
UMLS:ICD10CM:C15-C26	Malignant neoplasms of digestive organs
UMLS:ICD10CM:C40-C41	Malignant neoplasms of bone and articular cartilage
UMLS:ICD10CM:C43-C44	Melanoma and other malignant neoplasms of skin
UMLS:ICD10CM:C76-C80	Malignant neoplasms of ill-defined, other secondary and unspecified sites
UMLS:ICD10CM:C73-C75	Malignant neoplasms of thyroid and other endocrine glands
UMLS:ICD10CM:C69-C72	Malignant neoplasms of eye, brain and other parts of central nervous system
UMLS:ICD10CM:C64-C68	Malignant neoplasms of urinary tract
UMLS:ICD10CM:C60-C63	Malignant neoplasms of male genital organs
UMLS:ICD10CM:C51-C58	Malignant neoplasms of female genital organs
UMLS:ICD10CM:C7A-C7A	Malignant neuroendocrine tumors (C7A)
UMLS:ICD10CM:C7B-C7B	Secondary neuroendocrine tumors (C7B)
UMLS:ICD10CM:C81-C96	Malignant neoplasms of lymphoid, hematopoietic and related tissue
Definition for outcomes	
UMLS:ICD10CM:F01-F99	Mental, Behavioral and Neurodevelopmental disorders
UMLS:ICD10CM:G30	Alzheimer's disease

UMLS:ICD10CM:G30-G32	Other degenerative diseases of the nervous system
UMLS:ICD10CM:F01	Vascular dementia
UMLS:ICD10CM:F02	Dementia in other diseases classified elsewhere
UMLS:ICD10CM:F03	Unspecified dementia
UMLS:ICD10CM:F30-F39	Mood [affective] disorders
UMLS:ICD10CM:X71-X83	Intentional self-harm
UMLS:ICD10CM:S00-S09	Injuries to the head
UMLS:ICD10CM:S70-S79	Injuries to the hip and thigh
UMLS:ICD10CM:F40-F48	Anxiety, dissociative, stress-related, somatoform and other nonpsychotic mental disorders
UMLS:ICD10CM:F41	Other anxiety disorders
UMLS:ICD10CM:G20-G26	Extrapyramidal and movement disorders

Journal of
Clinical Medicine

MDPI

Table S2. Demographic and clinical characteristics before and after matching between Cohort A and Cohort B.

Attribute	Before matching						After matching					
	Cohort A (tamsulosin)		Cohort B (doxazosin)				Cohort A (tamsulosin)		Cohort B (doxazosin)			
Patient numbers	N = 213,690		N = 20,517				N = 20,511		N = 20,511			
Demographics	N	%	N	%	p-value	SMD	N	%	N	%	p-value	SMD
Age												
Current Age	73.6 ± 11.6		75.9 ± 11.3		<0.001	0.193	76.5 ± 10.5		75.9 ± 11.3		<0.001	0.059
Age at Index	66.8 ± 11.9		67.3 ± 11.7		<0.001	0.041	68.0 ± 11.0		67.3 ± 11.7		<0.001	0.059
Ethnicity												
Not Hispanic or Latino	144,276	67.5%	13,847	67.5%	0.939	0.001	13,918	67.9%	13,844	67.5%	0.435	0.008
Hispanic or Latino	14,352	6.7%	1,444	7.0%	0.079	0.013	1,337	6.5%	1,443	7.0%	0.037	0.021
Unknown Ethnicity	55,062	25.8%	5,226	25.5%	0.355	0.007	5,256	25.6%	5,224	25.5%	0.717	0.004
Race												
White	151,669	71.0%	13,858	67.5%	<0.001	0.074	14,117	68.8%	13,858	67.6%	0.006	0.027
Asian	9,397	4.4%	763	3.7%	<0.001	0.034	709	3.5%	763	3.7%	0.152	0.014
Black or African American	24,384	11.4%	3,238	15.8%	<0.001	0.128	3,082	15.0%	3,233	15.8%	0.039	0.020
American Indian or Alaska Native	442	0.2%	49	0.2%	0.339	0.007	39	0.2%	49	0.2%	0.286	0.011
Native Hawaiian or Other Pacific Islander	673	0.3%	34	0.2%	<0.001	0.030	49	0.2%	34	0.2%	0.099	0.016
Other Race	6,868	3.2%	555	2.7%	<0.001	0.030	503	2.5%	555	2.7%	0.105	0.016
Unknown Race	20,257	9.5%	2,020	9.8%	0.088	0.012	2,012	9.8%	2,019	9.8%	0.908	0.001
Marital Status												
Married	83,131	38.9%	7,647	37.3%	<0.001	0.034	7,773	37.9%	7,645	37.3%	0.192	0.013

Widowed	14,852	7.0%	1,452	7.1%	0.495	0.005	1,499	7.3%	1,451	7.1%	0.359	0.009
Never Married	17,064	8.0%	1,694	8.3%	0.172	0.010	1,630	7.9%	1,693	8.3%	0.254	0.011
Divorced	8,610	4.0%	781	3.8%	0.121	0.011	680	3.3%	781	3.8%	0.007	0.027
Legally Separated	989	0.5%	102	0.5%	0.490	0.005	90	0.4%	102	0.5%	0.385	0.009
Domestic partner	543	0.3%	41	0.2%	0.137	0.011	32	0.2%	41	0.2%	0.292	0.010
Diagnosis												
Metabolic disorders	83,412	39.0%	8,402	41.0%	<0.001	0.039	7,860	38.3%	8,401	41.0%	<0.001	0.054
Hypertensive diseases	85,497	40.0%	10,111	49.3%	<0.001	0.187	9,641	47.0%	10,105	49.3%	<0.001	0.045
Diabetes mellitus	42,070	19.7%	4,800	23.4%	<0.001	0.090	4,362	21.3%	4,798	23.4%	<0.001	0.051
Diseases of arteries, arterioles and capillaries	19,179	9.0%	1,973	9.6%	0.002	0.022	1,803	8.8%	1,972	9.6%	0.004	0.029
Overweight, obesity and other hyperalimentation	22,424	10.5%	2,250	11.0%	0.035	0.015	2,019	9.8%	2,250	11.0%	<0.001	0.037
Cerebrovascular diseases	14,423	6.7%	1,730	8.4%	<0.001	0.064	1,599	7.8%	1,728	8.4%	0.020	0.023
Other nutritional deficiencies	12,498	5.8%	1,202	5.9%	0.954	<0.001	1,049	5.1%	1,202	5.9%	0.001	0.033
Malnutrition	2,800	1.3%	282	1.4%	0.441	0.006	253	1.2%	282	1.4%	0.207	0.012
Nicotine dependence, unspecified, uncomplicated	0	0.0%	0	0.0%	--	--	0	0.0%	0	0.0%	--	--
Alcohol related disorders	0	0.0%	0	0.0%	--	--	0	0.0%	0	0.0%	--	--
BMI	29.5 ± 6.2		30.5 ± 6.4		<0.001	0.157	30.2 ± 6.2		30.5 ± 6.4		0.004	0.048
0 - 18.50 kg/m2	2,679	1.3%	193	0.9%	<0.001	0.030	184	0.9%	193	0.9%	0.641	0.005
18.50 - 25 kg/m2	24,432	11.4%	1,628	7.9%	<0.001	0.119	1,519	7.4%	1,628	7.9%	0.043	0.020
25 - 30 kg/m2	45,084	21.1%	3,583	17.5%	<0.001	0.092	3,210	15.7%	3,583	17.5%	<0.001	0.049
30 - 0 kg/m2	43,855	20.5%	4,126	20.1%	0.162	0.010	3,820	18.6%	4,126	20.1%	<0.001	0.038

Table S3. Demographic and clinical characteristics before and after matching between Cohort A and Cohort C.

Attribute	Before matching						After matching					
	Cohort A (tamsulosin)		Cohort C (terazosin)				Cohort A (tamsulosin)		Cohort C (terazosin)			
Patient numbers	N = 213,690		N = 15,448				N = 15,446		N = 15,446			
Demographics	N	%	N	%	p-value	SMD	N	%	N	%	p-value	SMD
Age												
Current Age	73.6 ± 11.6		78.0 ± 10.7		<0.001	0.387	76.5 ± 10.5		78.0 ± 10.7		0.002	0.035
Age at Index	66.8 ± 11.9		69.1 ± 11.5		<0.001	0.195	68.0 ± 11.0		69.1 ± 11.5		<0.001	0.043
Ethnicity												
Not Hispanic or Latino	144,276	67.5%	10,701	69.3%	<0.001	0.038	13,918	67.9%	10,700	69.3%	0.205	0.014
Hispanic or Latino	14,352	6.7%	808	5.2%	<0.001	0.063	1,337	6.5%	808	5.2%	0.146	0.017
Unknown Ethnicity	55,062	25.8%	3,939	25.5%	0.461	0.006	5,256	25.6%	3,938	25.5%	0.039	0.023
Race												
White	151,669	71.0%	11,198	72.5%	<0.001	0.034	14,117	68.8%	11,197	72.5%	<0.001	0.040
Asian	9,397	4.4%	576	3.7%	<0.001	0.034	709	3.5%	576	3.7%	0.006	0.031
Black or African American	24,384	11.4%	1,810	11.7%	0.249	0.010	3,082	15.0%	1,810	11.7%	0.002	0.035
American Indian or Alaska Native	442	0.2%	23	0.1%	0.122	0.014	39	0.2%	23	0.1%	0.668	0.005
Native Hawaiian or Other Pacific Islander	673	0.3%	86	0.6%	<0.001	0.037	49	0.2%	85	0.6%	0.261	0.013
Other Race	6,868	3.2%	356	2.3%	<0.001	0.056	503	2.5%	356	2.3%	0.790	0.003
Unknown Race	20,257	9.5%	1,399	9.1%	0.082	0.015	2,012	9.8%	1,399	9.1%	1.000	<0.001
Marital Status												
Married	83,131	38.9%	5,951	38.5%	0.350	0.008	7,773	37.9%	5,950	38.5%	0.174	0.015

Widowed	14,852	7.0%	1,285	8.3%	<0.001	0.052	1,499	7.3%	1,285	8.3%	0.869	0.002
Never Married	17,064	8.0%	1,118	7.2%	0.001	0.028	1,630	7.9%	1,118	7.2%	0.175	0.015
Divorced	8,610	4.0%	629	4.1%	0.795	0.002	680	3.3%	629	4.1%	0.219	0.014
Legally Separated	989	0.5%	50	0.3%	0.013	0.022	90	0.4%	50	0.3%	0.535	0.007
Domestic partner	543	0.3%	45	0.3%	0.378	0.007	32	0.2%	45	0.3%	0.108	0.018
Diagnosis												
Metabolic disorders	83,412	39.0%	5,590	36.2%	<0.001	0.059	7,860	38.3%	5,590	36.2%	<0.001	0.047
Hypertensive diseases	85,497	40.0%	6,691	43.3%	<0.001	0.067	9,641	47.0%	6,689	43.3%	<0.001	0.053
Diabetes mellitus	42,070	19.7%	2,947	19.1%	0.065	0.015	4,362	21.3%	2,946	19.1%	<0.001	0.062
Diseases of arteries, arterioles and capillaries	19,179	9.0%	1,225	7.9%	<0.001	0.038	1,803	8.8%	1,225	7.9%	0.007	0.031
Overweight, obesity and other hyperalimentation	22,424	10.5%	1,377	8.9%	<0.001	0.053	2,019	9.8%	1,376	8.9%	<0.001	0.044
Cerebrovascular diseases	14,423	6.7%	1,073	6.9%	0.348	0.008	1,599	7.8%	1,072	6.9%	0.053	0.022
Other nutritional deficiencies	12,498	5.8%	732	4.7%	<0.001	0.050	1,049	5.1%	732	4.7%	<0.001	0.041
Malnutrition	2,800	1.3%	138	0.9%	<0.001	0.040	253	1.2%	138	0.9%	0.904	0.001
Nicotine dependence, unspecified, uncomplicated	0	0.0%	0	0.0%	--	--	0	0.0%	0	0.0%	--	--
Alcohol related disorders	0	0.0%	0	0.0%	--	--	0	0.0%	0	0.0%	--	--
BMI	29.5 ± 6.2		30.3 ± 6.1		<0.001	0.125	30.2 ± 6.2		30.3 ± 6.1		<0.001	0.076
0 - 18.50 kg/m2	2,679	1.3%	94	0.6%	<0.001	0.067	184	0.9%	94	0.6%	0.145	0.017
18.50 - 25 kg/m2	24,432	11.4%	1,102	7.1%	<0.001	0.149	1,519	7.4%	1,102	7.1%	0.254	0.013
25 - 30 kg/m2	45,084	21.1%	2,417	15.6%	<0.001	0.141	3,210	15.7%	2,417	15.6%	0.104	0.019
30 - 0 kg/m2	43,855	20.5%	2,669	17.3%	<0.001	0.083	3,820	18.6%	2,669	17.3%	0.013	0.028

Table S4. Demographic and clinical characteristics before and after matching between Cohort A and Cohort D.

Attribute	Before matching						After matching					
	Cohort A (tamsulosin)		Cohort D (alfuzosin)				Cohort A (tamsulosin)		Cohort D (alfuzosin)			
Patient numbers	N = 213,690		N = 10,138				N = 10,137		N = 10,137			
Demographics	N	%	N	%	p-value	SMD	N	%	N	%	p-value	SMD
Age												
Current Age	73.6 ± 11.6		71.3 ± 11.8		<0.001	0.196	76.5 ± 10.5		71.4 ± 11.8		0.650	0.006
Age at Index	66.8 ± 11.9		64.1 ± 11.6		<0.001	0.228	68.0 ± 11.0		64.1 ± 11.6		0.748	0.005
Ethnicity												
Not Hispanic or Latino	144,276	67.5%	6,665	65.7%	<0.001	0.038	13,918	67.9%	6,664	65.7%	0.247	0.016
Hispanic or Latino	14,352	6.7%	521	5.1%	<0.001	0.067	1,337	6.5%	521	5.1%	0.924	0.001
Unknown Ethnicity	55,062	25.8%	2,952	29.1%	<0.001	0.075	5,256	25.6%	2,952	29.1%	0.209	0.018
Race												
White	151,669	71.0%	7,448	73.5%	<0.001	0.056	14,117	68.8%	7,448	73.5%	<0.001	0.051
Asian	9,397	4.4%	394	3.9%	0.014	0.026	709	3.5%	394	3.9%	0.117	0.022
Black or African American	24,384	11.4%	806	8.0%	<0.001	0.117	3,082	15.0%	806	8.0%	0.171	0.019
American Indian or Alaska Native	442	0.2%	17	0.2%	0.394	0.009	39	0.2%	17	0.2%	0.423	0.011
Native Hawaiian or Other Pacific Islander	673	0.3%	21	0.2%	0.056	0.021	49	0.2%	21	0.2%	0.654	0.006
Other Race	6,868	3.2%	320	3.2%	0.748	0.003	503	2.5%	320	3.2%	0.369	0.013
Unknown Race	20,257	9.5%	1,132	11.2%	<0.001	0.055	2,012	9.8%	1,131	11.2%	0.008	0.038
Marital Status												
Married	83,131	38.9%	4,252	41.9%	<0.001	0.062	7,773	37.9%	4,252	41.9%	0.909	0.002

Widowed	14,852	7.0%	500	4.9%	<0.001	0.085	1,499	7.3%	500	4.9%	0.106	0.023
Never Married	17,064	8.0%	524	5.2%	<0.001	0.114	1,630	7.9%	524	5.2%	0.522	0.009
Divorced	8,610	4.0%	322	3.2%	<0.001	0.046	680	3.3%	322	3.2%	0.349	0.013
Legally Separated	989	0.5%	25	0.2%	0.002	0.036	90	0.4%	25	0.2%	0.781	0.004
Domestic partner	543	0.3%	20	0.2%	0.264	0.012	32	0.2%	20	0.2%	0.223	0.017
Diagnosis												
Metabolic disorders	83,412	39.0%	3,192	31.5%	<0.001	0.158	7,860	38.3%	3,192	31.5%	0.114	0.022
Hypertensive diseases	85,497	40.0%	2,928	28.9%	<0.001	0.236	9,641	47.0%	2,928	28.9%	0.768	0.004
Diabetes mellitus	42,070	19.7%	1,175	11.6%	<0.001	0.224	4,362	21.3%	1,175	11.6%	0.537	0.009
Diseases of arteries, arterioles and capillaries	19,179	9.0%	540	5.3%	<0.001	0.142	1,803	8.8%	540	5.3%	0.024	0.032
Overweight, obesity and other hyperalimentation	22,424	10.5%	761	7.5%	<0.001	0.105	2,019	9.8%	761	7.5%	0.185	0.019
Cerebrovascular diseases	14,423	6.7%	343	3.4%	<0.001	0.154	1,599	7.8%	343	3.4%	0.530	0.009
Other nutritional deficiencies	12,498	5.8%	562	5.5%	0.200	0.013	1,049	5.1%	562	5.5%	0.002	0.045
Malnutrition	2,800	1.3%	18	0.2%	<0.001	0.132	253	1.2%	18	0.2%	0.731	0.005
Nicotine dependence, unspecified, uncomplicated	0	0.0%	0	0.0%	--	--	0	0.0%	0	0.0%	--	--
Alcohol related disorders	0	0.0%	0	0.0%	--	--	0	0.0%	0	0.0%	--	--
BMI	29.5 ± 6.2		28.9 ± 5.2		<0.001	0.105	30.2 ± 6.2		28.9 ± 5.2		0.115	0.036
0 - 18.50 kg/m2	2,679	1.3%	97	1.0%	0.008	0.028	184	0.9%	97	1.0%	0.885	0.002
18.50 - 25 kg/m2	24,432	11.4%	1,105	10.9%	0.099	0.017	1,519	7.4%	1,104	10.9%	0.008	0.037
25 - 30 kg/m2	45,084	21.1%	2,199	21.7%	0.153	0.014	3,210	15.7%	2,198	21.7%	0.002	0.043
30 - 0 kg/m2	43,855	20.5%	1,684	16.6%	<0.001	0.101	3,820	18.6%	1,684	16.6%	0.177	0.019

Table S5. Demographic and clinical characteristics before and after matching between Cohort A and Cohort E.

Attribute	Before matching						After matching					
	Cohort A (tamsulosin)		Cohort E (silodosin)				Cohort A (tamsulosin)		Cohort E (silodosin)			
Patient numbers	N = 213,690		N = 3,145				N = 3,145		N = 3,145			
Demographics	N	%	N	%	p-value	SMD	N	%	N	%	p-value	SMD
Age												
Current Age	73.6 ± 11.6		73.1 ± 11.1		0.012	0.046	76.5 ± 10.5		73.1 ± 11.1		0.885	0.004
Age at Index	66.8 ± 11.9		66.0 ± 11.6		<0.001	0.068	68.0 ± 11.0		66.0 ± 11.6		0.872	0.004
Ethnicity												
Not Hispanic or Latino	144,276	67.5%	2,237	71.1%	<0.001	0.078	13,918	67.9%	2,237	71.1%	0.579	0.014
Hispanic or Latino	14,352	6.7%	159	5.1%	<0.001	0.071	1,337	6.5%	159	5.1%	0.732	0.009
Unknown Ethnicity	55,062	25.8%	749	23.8%	0.013	0.045	5,256	25.6%	749	23.8%	0.680	0.010
Race												
White	151,669	71.0%	2,314	73.6%	0.001	0.058	14,117	68.8%	2,314	73.6%	0.056	0.048
Asian	9,397	4.4%	135	4.3%	0.776	0.005	709	3.5%	135	4.3%	0.119	0.039
Black or African American	24,384	11.4%	246	7.8%	<0.001	0.122	3,082	15.0%	246	7.8%	0.925	0.002
American Indian or Alaska Native	442	0.2%	10	0.3%	0.175	0.022	39	0.2%	10	0.3%	0.002	0.080
Native Hawaiian or Other Pacific Islander	673	0.3%	10	0.3%	0.976	0.001	49	0.2%	10	0.3%	1.000	<0.001
Other Race	6,868	3.2%	77	2.4%	0.015	0.046	503	2.5%	77	2.4%	0.450	0.019
Unknown Race	20,257	9.5%	367	11.7%	<0.001	0.071	2,012	9.8%	367	11.7%	0.230	0.030
Marital Status												
Married	83,131	38.9%	1,502	47.8%	<0.001	0.179	7,773	37.9%	1,502	47.8%	0.631	0.012
Widowed	14,852	7.0%	215	6.8%	0.803	0.005	1,499	7.3%	215	6.8%	0.587	0.014

Never Married	17,064	8.0%	187	5.9%	<0.001	0.080	1,630	7.9%	187	5.9%	0.707	0.009
Divorced	8,610	4.0%	93	3.0%	0.002	0.058	680	3.3%	93	3.0%	0.356	0.023
Legally Separated	989	0.5%	10	0.3%	0.234	0.023	90	0.4%	10	0.3%	1.000	<0.001
Domestic partner	543	0.3%	10	0.3%	0.481	0.012	32	0.2%	10	0.3%	1.000	<0.001
Diagnosis												
Metabolic disorders	83,412	39.0%	1,017	32.3%	<0.001	0.140	7,860	38.3%	1,017	32.3%	0.465	0.018
Hypertensive diseases	85,497	40.0%	943	30.0%	<0.001	0.211	9,641	47.0%	943	30.0%	0.562	0.015
Diabetes mellitus	42,070	19.7%	391	12.4%	<0.001	0.199	4,362	21.3%	391	12.4%	0.353	0.023
Diseases of arteries, arterioles and capillaries	19,179	9.0%	201	6.4%	<0.001	0.097	1,803	8.8%	201	6.4%	0.795	0.007
Overweight, obesity and other hyperalimentation	22,424	10.5%	205	6.5%	<0.001	0.143	2,019	9.8%	205	6.5%	0.878	0.004
Cerebrovascular diseases	14,423	6.7%	173	5.5%	0.006	0.052	1,599	7.8%	173	5.5%	0.232	0.030
Other nutritional deficiencies	12,498	5.8%	193	6.1%	0.494	0.012	1,049	5.1%	193	6.1%	0.454	0.019
Malnutrition	2,800	1.3%	26	0.8%	0.018	0.047	253	1.2%	26	0.8%	0.776	0.007
Nicotine dependence, unspecified, uncomplicated	0	0.0%	0	0.0%	--	--	0	0.0%	0	0.0%	--	--
Alcohol related disorders	0	0.0%	0	0.0%	--	--	0	0.0%	0	0.0%	--	--
BMI	29.5 ± 6.2		28.5 ± 5.4		<0.001	0.173	30.2 ± 6.2		28.5 ± 5.4		0.245	0.048
0 - 18.50 kg/m2	2,679	1.3%	31	1.0%	0.179	0.025	184	0.9%	31	1.0%	0.795	0.007
18.50 - 25 kg/m2	24,432	11.4%	359	11.4%	0.974	0.001	1,519	7.4%	359	11.4%	0.123	0.039
25 - 30 kg/m2	45,084	21.1%	677	21.5%	0.559	0.010	3,210	15.7%	677	21.5%	0.066	0.046
30 - 0 kg/m2	43,855	20.5%	464	14.8%	<0.001	0.152	3,820	18.6%	464	14.8%	0.591	0.014

Table S6. Demographic and clinical characteristics before and after matching between Cohort A and Cohort F.

Attribute	Before matching						After matching					
	Cohort A (tamsulosin)		Cohort F (prazosin)				Cohort A (tamsulosin)		Cohort F (prazosin)			
Patient numbers	N = 213,690		N = 1,184				N = 1,184		N = 1,184			
Demographics	N	%	N	%	p-value	SMD	N	%	N	%	p-value	SMD
Age												
Current Age	73.6 ± 11.6		68.4 ± 15.0		<0.001	0.392	76.5 ± 10.5		68.4 ± 15.0		0.847	0.008
Age at Index	66.8 ± 11.9		60.8 ± 14.7		<0.001	0.45	68.0 ± 11.0		60.8 ± 14.7		0.906	0.005
Ethnicity												
Not Hispanic or Latino	144,276	67.5%	824	69.6%	0.128	0.045	13,918	67.9%	824	69.6%	0.894	0.006
Hispanic or Latino	14,352	6.7%	79	6.7%	0.952	0.002	1,337	6.5%	79	6.7%	0.739	0.014
Unknown Ethnicity	55,062	25.8%	281	23.7%	0.110	0.047	5,256	25.6%	281	23.7%	0.736	0.014
Race												
White	151,669	71.0%	796	67.2%	0.005	0.081	14,117	68.8%	796	67.2%	0.568	0.023
Asian	9,397	4.4%	41	3.5%	0.118	0.048	709	3.5%	41	3.5%	0.345	0.039
Black or African American	24,384	11.4%	200	16.9%	<0.001	0.158	3,082	15.0%	200	16.9%	0.699	0.016
American Indian or Alaska Native	442	0.2%	10	0.8%	<0.001	0.088	39	0.2%	10	0.8%	1.000	<0.001
Native Hawaiian or Other Pacific Islander	673	0.3%	10	0.8%	0.001	0.070	49	0.2%	10	0.8%	1.000	<0.001
Other Race	6,868	3.2%	32	2.7%	0.320	0.030	503	2.5%	32	2.7%	0.898	0.005
Unknown Race	20,257	9.5%	108	9.1%	0.675	0.012	2,012	9.8%	108	9.1%	0.887	0.006
Marital Status												
Married	83,131	38.9%	380	32.1%	<0.001	0.143	7,773	37.9%	380	32.1%	0.895	0.005

Widowed	14,852	7.0%	76	6.4%	0.473	0.021	1,499	7.3%	76	6.4%	0.416	0.033
Never Married	17,064	8.0%	168	14.2%	<0.001	0.199	1,630	7.9%	168	14.2%	0.815	0.010
Divorced	8,610	4.0%	57	4.8%	0.171	0.038	680	3.3%	57	4.8%	0.923	0.004
Legally Separated	989	0.5%	10	0.8%	0.054	0.047	90	0.4%	10	0.8%	1.000	<0.001
Domestic partner	543	0.3%	10	0.8%	<0.001	0.080	32	0.2%	10	0.8%	1.000	<0.001
Diagnosis												
Metabolic disorders	83,412	39.0%	358	30.2%	<0.001	0.186	7,860	38.3%	358	30.2%	0.500	0.028
Hypertensive diseases	85,497	40.0%	446	37.7%	0.101	0.048	9,641	47.0%	446	37.7%	0.095	0.069
Diabetes mellitus	42,070	19.7%	206	17.4%	0.048	0.059	4,362	21.3%	206	17.4%	0.623	0.020
Diseases of arteries, arterioles and capillaries	19,179	9.0%	95	8.0%	0.253	0.034	1,803	8.8%	95	8.0%	0.702	0.016
Overweight, obesity and other hyperalimentation	22,424	10.5%	125	10.6%	0.943	0.002	2,019	9.8%	125	10.6%	0.736	0.014
Cerebrovascular diseases	14,423	6.7%	76	6.4%	0.651	0.013	1,599	7.8%	76	6.4%	0.803	0.010
Other nutritional deficiencies	12,498	5.8%	53	4.5%	0.045	0.062	1,049	5.1%	53	4.5%	0.472	0.030
Malnutrition	2,800	1.3%	14	1.2%	0.700	0.012	253	1.2%	14	1.2%	0.412	0.034
Nicotine dependence, unspecified, uncomplicated	0	0.0%	0	0.0%	--	--	0	0.0%	0	0.0%	--	--
Alcohol related disorders	0	0.0%	0	0.0%	--	--	0	0.0%	0	0.0%	--	--
BMI	29.5 ± 6.2		30.9 ± 6.8		<0.001	0.212	30.2 ± 6.2		30.9 ± 6.8		0.950	0.004
0 - 18.50 kg/m2	2,679	1.3%	13	1.1%	0.631	0.014	184	0.9%	13	1.1%	0.530	0.026
18.50 - 25 kg/m2	24,432	11.4%	93	7.9%	<0.001	0.121	1,519	7.4%	93	7.9%	0.388	0.036
25 - 30 kg/m2	45,084	21.1%	186	15.7%	<0.001	0.139	3,210	15.7%	186	15.7%	0.822	0.009
30 - 0 kg/m2	43,855	20.5%	232	19.6%	0.430	0.023	3,820	18.6%	232	19.6%	0.719	0.015

Table S7. Demographic and clinical characteristics before and after matching between Cohort G and Cohort H.

Attribute	Before matching						After matching					
	Cohort G (tamsulosin)		Cohort H (doxazosin)				Cohort G (tamsulosin)		Cohort H (doxazosin)			
Patient numbers	N = 184,381		N = 10,913				N = 10,910		N = 10,910			
Demographics	N	%	N	%	p-value	SMD	N	%	N	%	p-value	SMD
Age												
Current Age	73.6 ± 11.6		75.6 ± 11.6		<0.001	0.171	76.5 ± 10.5		75.6 ± 11.6		<0.001	0.081
Age at Index	66.9 ± 12.0		67.1 ± 12.0		0.033	0.021	68.0 ± 10.9		67.1 ± 12.0		<0.001	0.08
Ethnicity												
Not Hispanic or Latino	128,260	69.6%	7,503	68.8%	0.074	0.018	7,603	69.7%	7,501	68.8%	0.135	0.020
Hispanic or Latino	12,912	7.0%	819	7.5%	0.046	0.019	721	6.6%	818	7.5%	0.010	0.035
Unknown Ethnicity	43,209	23.4%	2,591	23.7%	0.461	0.007	2,586	23.7%	2,591	23.7%	0.937	0.001
Race												
White	131,275	71.2%	7,373	67.6%	<0.001	0.079	7,584	69.5%	7,373	67.6%	0.002	0.042
Asian	8,332	4.5%	416	3.8%	0.001	0.035	385	3.5%	416	3.8%	0.264	0.015
Black or African American	19,842	10.8%	1,632	15.0%	<0.001	0.126	1,526	14.0%	1,630	14.9%	0.045	0.027
American Indian or Alaska Native	373	0.2%	31	0.3%	0.068	0.017	28	0.3%	31	0.3%	0.696	0.005
Native Hawaiian or Other Pacific Islander	615	0.3%	19	0.2%	0.004	0.032	18	0.2%	19	0.2%	0.869	0.002
Other Race	6,130	3.3%	309	2.8%	0.005	0.029	261	2.4%	309	2.8%	0.042	0.028
Unknown Race	17,814	9.7%	1,133	10.4%	0.013	0.024	1,108	10.2%	1,132	10.4%	0.592	0.007
Marital Status												
Married	70,100	38.0%	3,910	35.8%	<0.001	0.045	3,950	36.2%	3,910	35.8%	0.573	0.008

Widowed	12,627	6.8%	685	6.3%	0.021	0.023	709	6.5%	685	6.3%	0.506	0.009
Never Married	14,305	7.8%	887	8.1%	0.161	0.014	782	7.2%	887	8.1%	0.007	0.036
Divorced	7,255	3.9%	379	3.5%	0.016	0.024	346	3.2%	379	3.5%	0.213	0.017
Legally Separated	819	0.4%	50	0.5%	0.831	0.002	41	0.4%	50	0.5%	0.344	0.013
Domestic partner	473	0.3%	22	0.2%	0.267	0.011	19	0.2%	22	0.2%	0.639	0.006
Diagnosis												
Metabolic disorders	72,025	39.1%	4,266	39.1%	0.954	0.001	3,985	36.5%	4,265	39.1%	<0.001	0.053
Hypertensive diseases	73,358	39.8%	5,232	47.9%	<0.001	0.165	4,921	45.1%	5,229	47.9%	<0.001	0.057
Diabetes mellitus	36,235	19.7%	2,417	22.1%	<0.001	0.061	2,190	20.1%	2,417	22.2%	<0.001	0.051
Diseases of arteries, arterioles and capillaries	16,359	8.9%	941	8.6%	0.372	0.009	834	7.6%	941	8.6%	0.008	0.036
Overweight, obesity and other hyperalimentation	19,490	10.6%	1,153	10.6%	0.987	<0.001	1,055	9.7%	1,153	10.6%	0.028	0.030
Cerebrovascular diseases	12,254	6.6%	791	7.2%	0.014	0.024	707	6.5%	790	7.2%	0.026	0.030
Other nutritional deficiencies	10,780	5.8%	612	5.6%	0.301	0.010	549	5.0%	612	5.6%	0.057	0.026
Malnutrition	2,446	1.3%	121	1.1%	0.052	0.020	96	0.9%	121	1.1%	0.088	0.023
Nicotine dependence, unspecified, uncomplicated	0	0.0%	0	0.0%	--	--	0	0.0%	0	0.0%	--	--
Alcohol related disorders	0	0.0%	0	0.0%	--	--	0	0.0%	0	0.0%	--	--
BMI	29.5 ± 6.2		30.6 ± 6.4		<0.001	0.170	30.2 ± 6.2		30.6 ± 6.4		0.009	0.061
0 - 18.50 kg/m2	2,453	1.3%	95	0.9%	<0.001	0.044	81	0.7%	95	0.9%	0.289	0.014
18.50 - 25 kg/m2	21,666	11.8%	747	6.8%	<0.001	0.170	722	6.6%	747	6.8%	0.499	0.009
25 - 30 kg/m2	39,691	21.5%	1,795	16.4%	<0.001	0.130	1,660	15.2%	1,795	16.5%	0.012	0.034
30 - 0 kg/m2	38,650	21.0%	2,087	19.1%	<0.001	0.046	1,939	17.8%	2,087	19.1%	0.010	0.035

Table S8. Demographic and clinical characteristics before and after matching between Cohort G and Cohort I.

Attribute	Before matching						After matching					
	Cohort G (tamsulosin)		Cohort I (terazosin)				Cohort G (tamsulosin)		Cohort I (terazosin)			
Patient numbers	N = 184,381		N = 8,487				N = 8,484		N = 8,484			
Demographics	N	%	N	%	p-value	SMD	N	%	N	%	p-value	SMD
Age												
Current Age	73.6 ± 11.6		78.2 ± 10.7		<0.001	0.418	76.5 ± 10.5		78.2 ± 10.7		0.069	0.028
Age at Index	66.9 ± 12.0		69.6 ± 11.5		<0.001	0.232	68.0 ± 10.9		69.6 ± 11.5		0.018	0.036
Ethnicity												
Not Hispanic or Latino	128,260	69.6%	5,777	68.1%	0.003	0.032	7,603	69.7%	5,776	68.1%	0.393	0.013
Hispanic or Latino	12,912	7.0%	452	5.3%	<0.001	0.070	721	6.6%	452	5.3%	0.237	0.018
Unknown Ethnicity	43,209	23.4%	2,258	26.6%	<0.001	0.073	2,586	23.7%	2,256	26.6%	0.137	0.023
Race												
White	131,275	71.2%	6,162	72.6%	0.005	0.031	7,584	69.5%	6,160	72.6%	0.030	0.033
Asian	8,332	4.5%	321	3.8%	0.001	0.037	385	3.5%	321	3.8%	0.045	0.031
Black or African American	19,842	10.8%	939	11.1%	0.379	0.010	1,526	14.0%	939	11.1%	0.032	0.033
American Indian or Alaska Native	373	0.2%	11	0.1%	0.142	0.018	28	0.3%	11	0.1%	0.548	0.009
Native Hawaiian or Other Pacific Islander	615	0.3%	43	0.5%	0.007	0.027	18	0.2%	42	0.5%	0.052	0.030
Other Race	6,130	3.3%	213	2.5%	<0.001	0.048	261	2.4%	213	2.5%	0.271	0.017
Unknown Race	17,814	9.7%	798	9.4%	0.430	0.009	1,108	10.2%	798	9.4%	0.979	<0.001
Marital Status												
Married	70,100	38.0%	3,255	38.4%	0.536	0.007	3,950	36.2%	3,253	38.3%	0.384	0.013
Widowed	12,627	6.8%	699	8.2%	<0.001	0.053	709	6.5%	699	8.2%	0.153	0.022

Never Married	14,305	7.8%	621	7.3%	0.137	0.017	782	7.2%	621	7.3%	0.037	0.032
Divorced	7,255	3.9%	326	3.8%	0.664	0.005	346	3.2%	326	3.8%	0.002	0.048
Legally Separated	819	0.4%	25	0.3%	0.041	0.025	41	0.4%	25	0.3%	0.365	0.014
Domestic partner	473	0.3%	29	0.3%	0.132	0.016	19	0.2%	29	0.3%	0.257	0.017
Diagnosis												
Metabolic disorders	72,025	39.1%	2,812	33.1%	<0.001	0.124	3,985	36.5%	2,812	33.1%	0.025	0.035
Hypertensive diseases	73,358	39.8%	3,466	40.8%	0.053	0.021	4,921	45.1%	3,463	40.8%	<0.001	0.054
Diabetes mellitus	36,235	19.7%	1,479	17.4%	<0.001	0.057	2,190	20.1%	1,478	17.4%	<0.001	0.056
Diseases of arteries, arterioles and capillaries	16,359	8.9%	617	7.3%	<0.001	0.059	834	7.6%	617	7.3%	0.008	0.041
Overweight, obesity and other hyperalimentation	19,490	10.6%	671	7.9%	<0.001	0.092	1,055	9.7%	670	7.9%	0.004	0.045
Cerebrovascular diseases	12,254	6.6%	519	6.1%	0.055	0.022	707	6.5%	518	6.1%	<0.001	0.054
Other nutritional deficiencies	10,780	5.8%	354	4.2%	<0.001	0.077	549	5.0%	354	4.2%	0.105	0.025
Malnutrition	2,446	1.3%	57	0.7%	<0.001	0.066	96	0.9%	57	0.7%	0.562	0.009
Nicotine dependence, unspecified, uncomplicated	0	0.0%	0	0.0%	--	--	0	0.0%	0	0.0%	--	--
Alcohol related disorders	0	0.0%	0	0.0%	--	--	0	0.0%	0	0.0%	--	--
BMI	29.5 ± 6.2		30.5 ± 6.2		<0.001	0.151	30.2 ± 6.2		30.5 ± 6.2		0.001	0.094
0 - 18.50 kg/m2	2,453	1.3%	50	0.6%	<0.001	0.076	81	0.7%	50	0.6%	0.921	0.002
18.50 - 25 kg/m2	21,666	11.8%	533	6.3%	<0.001	0.192	722	6.6%	533	6.3%	0.799	0.004
25 - 30 kg/m2	39,691	21.5%	1,184	14.0%	<0.001	0.199	1,660	15.2%	1,184	14.0%	0.304	0.016
30 - 0 kg/m2	38,650	21.0%	1,368	16.1%	<0.001	0.125	1,939	17.8%	1,368	16.1%	0.011	0.039

Table S9. Demographic and clinical characteristics before and after matching between Cohort G and Cohort J.

Attribute	Before matching						After matching					
	Cohort G (tamsulosin)		Cohort J (alfuzosin)				Cohort G (tamsulosin)		Cohort J (alfuzosin)			
Patient numbers	N = 184,381		N = 4,641				N = 4,641		N = 4,641			
Demographics	N	%	N	%	p-value	SMD	N	%	N	%	p-value	SMD
Age												
Current Age	73.6 ± 11.6		70.1 ± 12.0		<0.001	0.292	76.5 ± 10.5		70.1 ± 12.0		0.972	0.001
Age at Index	66.9 ± 12.0		63.2 ± 11.8		<0.001	0.309	68.0 ± 10.9		63.2 ± 11.8		0.866	0.004
Ethnicity												
Not Hispanic or Latino	128,260	69.6%	3,024	65.2%	<0.001	0.094	7,603	69.7%	3,024	65.2%	0.600	0.011
Hispanic or Latino	12,912	7.0%	258	5.6%	<0.001	0.060	721	6.6%	258	5.6%	0.434	0.016
Unknown Ethnicity	43,209	23.4%	1,359	29.3%	<0.001	0.133	2,586	23.7%	1,359	29.3%	0.873	0.003
Race												
White	131,275	71.2%	3,378	72.8%	0.018	0.035	7,584	69.5%	3,378	72.8%	0.454	0.016
Asian	8,332	4.5%	169	3.6%	0.004	0.044	385	3.5%	169	3.6%	0.306	0.021
Black or African American	19,842	10.8%	322	6.9%	<0.001	0.135	1,526	14.0%	322	6.9%	0.711	0.008
American Indian or Alaska Native	373	0.2%	10	0.2%	0.844	0.003	28	0.3%	10	0.2%	1.000	<0.001
Native Hawaiian or Other Pacific Islander	615	0.3%	12	0.3%	0.380	0.014	18	0.2%	12	0.3%	0.841	0.004
Other Race	6,130	3.3%	164	3.5%	0.433	0.011	261	2.4%	164	3.5%	0.738	0.007
Unknown Race	17,814	9.7%	588	12.7%	<0.001	0.096	1,108	10.2%	588	12.7%	0.707	0.008
Marital Status												
Married	70,100	38.0%	1,876	40.4%	0.001	0.049	3,950	36.2%	1,876	40.4%	0.146	0.030
Widowed	12,627	6.8%	170	3.7%	<0.001	0.143	709	6.5%	170	3.7%	0.912	0.002

Never Married	14,305	7.8%	224	4.8%	<0.001	0.121	782	7.2%	224	4.8%	0.885	0.003
Divorced	7,255	3.9%	132	2.8%	<0.001	0.060	346	3.2%	132	2.8%	0.568	0.012
Legally Separated	819	0.4%	11	0.2%	0.035	0.036	41	0.4%	11	0.2%	0.432	0.016
Domestic partner	473	0.3%	10	0.2%	0.584	0.008	19	0.2%	10	0.2%	1.000	<0.001
Diagnosis												
Metabolic disorders	72,025	39.1%	1,183	25.5%	<0.001	0.293	3,985	36.5%	1,183	25.5%	0.943	0.001
Hypertensive diseases	73,358	39.8%	1,086	23.4%	<0.001	0.358	4,921	45.1%	1,086	23.4%	0.494	0.014
Diabetes mellitus	36,235	19.7%	426	9.2%	<0.001	0.302	2,190	20.1%	426	9.2%	0.204	0.026
Diseases of arteries, arterioles and capillaries	16,359	8.9%	180	3.9%	<0.001	0.205	834	7.6%	180	3.9%	0.745	0.007
Overweight, obesity and other hyperalimentation	19,490	10.6%	297	6.4%	<0.001	0.150	1,055	9.7%	297	6.4%	0.548	0.012
Cerebrovascular diseases	12,254	6.6%	98	2.1%	<0.001	0.223	707	6.5%	98	2.1%	0.459	0.015
Other nutritional deficiencies	10,780	5.8%	200	4.3%	<0.001	0.070	549	5.0%	200	4.3%	0.534	0.013
Malnutrition	2,446	1.3%	10	0.2%	<0.001	0.127	96	0.9%	10	0.2%	1.000	<0.001
Nicotine dependence, unspecified, uncomplicated	0	0.0%	0	0.0%	--	--	0	0.0%	0	0.0%	--	--
Alcohol related disorders	0	0.0%	0	0.0%	--	--	0	0.0%	0	0.0%	--	--
BMI	29.5 ± 6.2		28.9 ± 5.1		<0.001	0.114	30.2 ± 6.2		28.9 ± 5.1		0.387	0.031
0 - 18.50 kg/m2	2,453	1.3%	51	1.1%	0.173	0.021	81	0.7%	51	1.1%	0.049	0.041
18.50 - 25 kg/m2	21,666	11.8%	443	9.5%	<0.001	0.072	722	6.6%	443	9.5%	0.593	0.011
25 - 30 kg/m2	39,691	21.5%	875	18.9%	<0.001	0.067	1,660	15.2%	875	18.9%	0.690	0.008
30 - 0 kg/m2	38,650	21.0%	648	14.0%	<0.001	0.185	1,939	17.8%	648	14.0%	0.397	0.018

Table S10. Demographic and clinical characteristics before and after matching between Cohort G and Cohort K.

Attribute	Before matching								After matching							
	Cohort G (tamsulosin)		Cohort K (silodosin)						Cohort G (tamsulosin)		Cohort K (silodosin)					
Patient numbers	N = 184,381		N = 1,061						N = 1,061		N = 1,061					
Demographics	N	%	N	%	p-value	SMD			N	%	N	%	p-value	SMD		
Age																
Current Age	73.6 ± 11.6		72.7 ± 11.2		0.011	0.080			76.5 ± 10.5		72.7 ± 11.2		0.825	0.010		
Age at Index	66.9 ± 12.0		65.7 ± 11.7		0.002	0.097			68.0 ± 10.9		65.7 ± 11.7		0.994	<0.001		
Ethnicity																
Not Hispanic or Latino	128,260	69.6%	721	68.0%	0.256	0.035			7,603	69.7%	721	68.0%	0.853	0.008		
Hispanic or Latino	12,912	7.0%	60	5.7%	0.086	0.055			721	6.6%	60	5.7%	1.000	<0.001		
Unknown Ethnicity	43,209	23.4%	280	26.4%	0.023	0.068			2,586	23.7%	280	26.4%	0.844	0.009		
Race																
White	131,275	71.2%	768	72.4%	0.395	0.026			7,584	69.5%	768	72.4%	0.139	0.064		
Asian	8,332	4.5%	54	5.1%	0.372	0.027			385	3.5%	54	5.1%	0.698	0.017		
Black or African American	19,842	10.8%	66	6.2%	<0.001	0.163			1,526	14.0%	66	6.2%	0.715	0.016		
American Indian or Alaska Native	373	0.2%	10	0.9%	<0.001	0.098			28	0.3%	10	0.9%	1.000	<0.001		
Native Hawaiian or Other Pacific Islander	615	0.3%	10	0.9%	0.001	0.077			18	0.2%	10	0.9%	0.002	0.138		
Other Race	6,130	3.3%	33	3.1%	0.698	0.012			261	2.4%	33	3.1%	0.133	0.065		
Unknown Race	17,814	9.7%	136	12.8%	0.001	0.100			1,108	10.2%	136	12.8%	0.286	0.046		
Marital Status																
Married	70,100	38.0%	493	46.5%	<0.001	0.172			3,950	36.2%	493	46.5%	0.433	0.034		

Widowed	12,627	6.8%	47	4.4%	0.002	0.105	709	6.5%	47	4.4%	0.177	0.059
Never Married	14,305	7.8%	51	4.8%	<0.001	0.122	782	7.2%	51	4.8%	0.288	0.046
Divorced	7,255	3.9%	27	2.5%	0.020	0.079	346	3.2%	27	2.5%	0.302	0.045
Legally Separated	819	0.4%	10	0.9%	0.015	0.060	41	0.4%	10	0.9%	0.002	0.138
Domestic partner	473	0.3%	10	0.9%	<0.001	0.089	19	0.2%	10	0.9%	1.000	<0.001
Diagnosis												
Metabolic disorders	72,025	39.1%	272	25.6%	<0.001	0.290	3,985	36.5%	272	25.6%	0.727	0.015
Hypertensive diseases	73,358	39.8%	251	23.7%	<0.001	0.352	4,921	45.1%	251	23.7%	0.799	0.011
Diabetes mellitus	36,235	19.7%	114	10.7%	<0.001	0.250	2,190	20.1%	114	10.7%	1.000	<0.001
Diseases of arteries, arterioles and capillaries	16,359	8.9%	42	4.0%	<0.001	0.202	834	7.6%	42	4.0%	0.518	0.028
Overweight, obesity and other hyperalimentation	19,490	10.6%	57	5.4%	<0.001	0.193	1,055	9.7%	57	5.4%	0.695	0.017
Cerebrovascular diseases	12,254	6.6%	43	4.1%	0.001	0.115	707	6.5%	43	4.1%	0.912	0.005
Other nutritional deficiencies	10,780	5.8%	48	4.5%	0.067	0.060	549	5.0%	48	4.5%	0.518	0.028
Malnutrition	2,446	1.3%	10	0.9%	0.275	0.036	96	0.9%	10	0.9%	1.000	<0.001
Nicotine dependence, unspecified, uncomplicated	0	0.0%	0	0.0%	--	--	0	0.0%	0	0.0%	--	--
Alcohol related disorders	0	0.0%	0	0.0%	--	--	0	0.0%	0	0.0%	--	--
BMI	29.5 ± 6.2		29.0 ± 5.6		0.111	0.091	30.2 ± 6.2		29.0 ± 5.6		0.564	0.045
0 - 18.50 kg/m2	2,453	1.3%	10	0.9%	0.271	0.037	81	0.7%	10	0.9%	1.000	<0.001
18.50 - 25 kg/m2	21,666	11.8%	92	8.7%	0.002	0.102	722	6.6%	92	8.7%	0.816	0.010
25 - 30 kg/m2	39,691	21.5%	174	16.4%	<0.001	0.131	1,660	15.2%	174	16.4%	0.636	0.021
30 - 0 kg/m2	38,650	21.0%	141	13.3%	<0.001	0.205	1,939	17.8%	141	13.3%	0.211	0.054

Table S11. Demographic and clinical characteristics before and after matching between Cohort G and Cohort L.

Attribute	Before matching						After matching					
	Cohort G (tamsulosin)		Cohort L (prazosin)				Cohort G (tamsulosin)		Cohort L (prazosin)			
Patient numbers	N = 184,381		N = 521				N = 521		N = 521			
Demographics	N	%	N	%	p-value	SMD	N	%	N	%	p-value	SMD
Age												
Current Age	73.6 ± 11.6		67.7 ± 16.5		<0.001	0.414	76.5 ± 10.5		67.7 ± 16.5		0.782	0.017
Age at Index	66.9 ± 12.0		60.2 ± 16.2		<0.001	0.466	68.0 ± 10.9		60.2 ± 16.2		0.778	0.017
Ethnicity												
Not Hispanic or Latino	128,260	69.6%	373	71.6%	0.314	0.045	7,603	69.7%	373	71.6%	0.784	0.017
Hispanic or Latino	12,912	7.0%	31	6.0%	0.347	0.043	721	6.6%	31	6.0%	1.000	<0.001
Unknown Ethnicity	43,209	23.4%	117	22.5%	0.599	0.023	2,586	23.7%	117	22.5%	0.768	0.018
Race												
White	131,275	71.2%	356	68.3%	0.149	0.062	7,584	69.5%	356	68.3%	0.947	0.004
Asian	8,332	4.5%	23	4.4%	0.909	0.005	385	3.5%	23	4.4%	0.333	0.060
Black or African American	19,842	10.8%	88	16.9%	<0.001	0.178	1,526	14.0%	88	16.9%	0.743	0.020
American Indian or Alaska Native	373	0.2%	10	1.9%	<0.001	0.168	28	0.3%	10	1.9%	1.000	<0.001
Native Hawaiian or Other Pacific Islander	615	0.3%	10	1.9%	<0.001	0.151	18	0.2%	10	1.9%	1.000	<0.001
Other Race	6,130	3.3%	17	3.3%	0.937	0.003	261	2.4%	17	3.3%	0.459	0.046
Unknown Race	17,814	9.7%	34	6.5%	0.016	0.115	1,108	10.2%	34	6.5%	0.401	0.052
Marital Status												
Married	70,100	38.0%	163	31.3%	0.002	0.142	3,950	36.2%	163	31.3%	0.500	0.042
Widowed	12,627	6.8%	29	5.6%	0.247	0.053	709	6.5%	29	5.6%	0.188	0.082

Never Married	14,305	7.8%	77	14.8%	<0.001	0.223	782	7.2%	77	14.8%	0.310	0.063
Divorced	7,255	3.9%	20	3.8%	0.910	0.005	346	3.2%	20	3.8%	0.870	0.010
Legally Separated	819	0.4%	10	1.9%	<0.001	0.137	41	0.4%	10	1.9%	0.001	0.198
Domestic partner	473	0.3%	10	1.9%	<0.001	0.161	19	0.2%	10	1.9%	0.001	0.198
Diagnosis												
Metabolic disorders	72,025	39.1%	161	30.9%	<0.001	0.172	3,985	36.5%	161	30.9%	0.788	0.017
Hypertensive diseases	73,358	39.8%	203	39.0%	0.702	0.017	4,921	45.1%	203	39.0%	0.656	0.028
Diabetes mellitus	36,235	19.7%	83	15.9%	0.033	0.097	2,190	20.1%	83	15.9%	1.000	<0.001
Diseases of arteries, arterioles and capillaries	16,359	8.9%	44	8.4%	0.732	0.015	834	7.6%	44	8.4%	0.734	0.021
Overweight, obesity and other hyperalimentation	19,490	10.6%	57	10.9%	0.784	0.012	1,055	9.7%	57	10.9%	0.921	0.006
Cerebrovascular diseases	12,254	6.6%	32	6.1%	0.645	0.021	707	6.5%	32	6.1%	0.799	0.016
Other nutritional deficiencies	10,780	5.8%	24	4.6%	0.228	0.056	549	5.0%	24	4.6%	0.881	0.009
Malnutrition	2,446	1.3%	10	1.9%	0.238	0.047	96	0.9%	10	1.9%	1.000	<0.001
Nicotine dependence, unspecified, uncomplicated	0	0.0%	0	0.0%	--	--	0	0.0%	0	0.0%	--	--
Alcohol related disorders	0	0.0%	0	0.0%	--	--	0	0.0%	0	0.0%	--	--
BMI	29.5 ± 6.2		30.2 ± 6.4		0.144	0.111	30.2 ± 6.2		30.2 ± 6.4		0.501	0.074
0 - 18.50 kg/m2	2,453	1.3%	10	1.9%	0.242	0.047	81	0.7%	10	1.9%	1.000	<0.001
18.50 - 25 kg/m2	21,666	11.8%	41	7.9%	0.006	0.131	722	6.6%	41	7.9%	0.176	0.084
25 - 30 kg/m2	39,691	21.5%	76	14.6%	<0.001	0.181	1,660	15.2%	76	14.6%	0.723	0.022
30 - 0 kg/m2	38,650	21.0%	87	16.7%	0.017	0.109	1,939	17.8%	87	16.7%	0.681	0.025

Table S12. Demographic and clinical characteristics before and after matching between Cohort M and Cohort N.

Attribute	Before matching						After matching					
	Cohort M (tamsulosin)		Cohort N (doxazosin)				Cohort M (tamsulosin)		Cohort N (doxazosin)			
Patient numbers	N = 234,573		N = 13,395				N = 13,392		N = 13,392			
Demographics	N	%	N	%	p-value	SMD	N	%	N	%	p-value	SMD
Age												
Current Age	74.3 ± 11.4		76.2 ± 11.4		<0.001	0.171	77.0 ± 10.4		76.2 ± 11.4		<0.001	0.071
Age at Index	67.6 ± 11.7		67.8 ± 11.8		0.054	0.017	68.5 ± 10.8		67.8 ± 11.8		<0.001	0.065
Ethnicity												
Not Hispanic or Latino	159,304	67.9%	8,903	66.5%	<0.001	0.031	8,984	67.1%	8,901	66.5%	0.282	0.013
Hispanic or Latino	14,927	6.4%	947	7.1%	0.001	0.028	851	6.4%	946	7.1%	0.020	0.028
Unknown Ethnicity	60,342	25.7%	3,545	26.5%	0.057	0.017	3,557	26.6%	3,545	26.5%	0.868	0.002
Race												
White	168,704	71.9%	9,129	68.2%	<0.001	0.082	9,445	70.5%	9,129	68.2%	<0.001	0.051
Asian	10,160	4.3%	478	3.6%	<0.001	0.039	424	3.2%	478	3.6%	0.067	0.022
Black or African American	25,669	10.9%	2,067	15.4%	<0.001	0.133	1,883	14.1%	2,065	15.4%	0.002	0.038
American Indian or Alaska Native	476	0.2%	33	0.2%	0.280	0.009	33	0.2%	33	0.2%	1.000	<0.001
Native Hawaiian or Other Pacific Islander	765	0.3%	24	0.2%	0.003	0.029	31	0.2%	24	0.2%	0.345	0.012
Other Race	7,251	3.1%	348	2.6%	0.001	0.030	292	2.2%	348	2.6%	0.025	0.027
Unknown Race	21,548	9.2%	1,316	9.8%	0.013	0.022	1,284	9.6%	1,315	9.8%	0.522	0.008
Marital Status												
Married	90,945	38.8%	4,962	37.0%	<0.001	0.036	5,138	38.4%	4,961	37.0%	0.026	0.027

Widowed	16,194	6.9%	919	6.9%	0.849	0.002	954	7.1%	919	6.9%	0.402	0.010
Never Married	17,989	7.7%	1,091	8.1%	0.044	0.018	1,000	7.5%	1,091	8.1%	0.038	0.025
Divorced	9,207	3.9%	460	3.4%	0.004	0.026	417	3.1%	460	3.4%	0.140	0.018
Legally Separated	1,028	0.4%	61	0.5%	0.770	0.003	41	0.3%	61	0.5%	0.047	0.024
Domestic partner	585	0.2%	30	0.2%	0.565	0.005	21	0.2%	30	0.2%	0.207	0.015
Diagnosis												
Metabolic disorders	97,105	41.4%	5,495	41.0%	0.393	0.008	5,217	39.0%	5,495	41.0%	0.001	0.042
Hypertensive diseases	98,698	42.1%	6,672	49.8%	<0.001	0.156	6,253	46.7%	6,669	49.8%	<0.001	0.062
Diabetes mellitus	47,422	20.2%	3,040	22.7%	<0.001	0.060	2,781	20.8%	3,040	22.7%	<0.001	0.047
Diseases of arteries, arterioles and capillaries	23,245	9.9%	1,266	9.5%	0.084	0.015	1,124	8.4%	1,266	9.5%	0.002	0.037
Overweight, obesity and other hyperalimentation	25,489	10.9%	1,453	10.8%	0.946	0.001	1,243	9.3%	1,453	10.8%	<0.001	0.052
Cerebrovascular diseases	16,484	7.0%	1,013	7.6%	0.019	0.021	911	6.8%	1,012	7.6%	0.017	0.029
Other nutritional deficiencies	14,768	6.3%	792	5.9%	0.075	0.016	685	5.1%	792	5.9%	0.004	0.035
Malnutrition	4,485	1.9%	193	1.4%	<0.001	0.037	164	1.2%	193	1.4%	0.122	0.019
Nicotine dependence, unspecified, uncomplicated	0	0.0%	0	0.0%	--	--	0	0.0%	0	0.0%	--	--
Alcohol related disorders	0	0.0%	0	0.0%	--	--	0	0.0%	0	0.0%	--	--
BMI	29.3 ± 6.1		30.4 ± 6.3		<0.001	0.179	30.0 ± 6.2		30.4 ± 6.3		0.007	0.057
0 - 18.50 kg/m2	3,382	1.4%	118	0.9%	<0.001	0.052	118	0.9%	118	0.9%	1.000	<0.001
18.50 - 25 kg/m2	30,259	12.9%	981	7.3%	<0.001	0.186	984	7.3%	981	7.3%	0.944	0.001
25 - 30 kg/m2	53,721	22.9%	2,253	16.8%	<0.001	0.153	2,068	15.4%	2,253	16.8%	0.002	0.038
30 - 0 kg/m2	49,753	21.2%	2,537	18.9%	<0.001	0.057	2,338	17.5%	2,537	18.9%	0.002	0.039

Table S13. Demographic and clinical characteristics before and after matching between Cohort M and Cohort O.

Attribute	Before matching						After matching					
	Cohort M (tamsulosin)		Cohort O (terazosin)				Cohort M (tamsulosin)		Cohort O (terazosin)			
Patient numbers	N = 234,573		N = 10,258				N = 10,258		N = 10,258			
Demographics	N	%	N	%	p-value	SMD	N	%	N	%	p-value	SMD
Age												
Current Age	74.3 ± 11.4		78.8 ± 10.5		<0.001	0.411	77.0 ± 10.4		78.8 ± 10.5		0.031	0.03
Age at Index	67.6 ± 11.7		70.2 ± 11.3		<0.001	0.227	68.5 ± 10.8		70.2 ± 11.3		0.004	0.04
Ethnicity												
Not Hispanic or Latino	159,304	67.9%	6,876	67.0%	0.061	0.019	8,984	67.1%	6,876	67.0%	0.678	0.006
Hispanic or Latino	14,927	6.4%	503	4.9%	<0.001	0.063	851	6.4%	503	4.9%	0.120	0.022
Unknown Ethnicity	60,342	25.7%	2,879	28.1%	<0.001	0.053	3,557	26.6%	2,879	28.1%	0.246	0.016
Race												
White	168,704	71.9%	7,496	73.1%	0.011	0.026	9,445	70.5%	7,496	73.1%	0.003	0.042
Asian	10,160	4.3%	388	3.8%	0.007	0.028	424	3.2%	388	3.8%	0.064	0.026
Black or African American	25,669	10.9%	1,138	11.1%	0.632	0.005	1,883	14.1%	1,138	11.1%	0.008	0.037
American Indian or Alaska Native	476	0.2%	14	0.1%	0.141	0.016	33	0.2%	14	0.1%	1.000	<0.001
Native Hawaiian or Other Pacific Islander	765	0.3%	51	0.5%	0.003	0.027	31	0.2%	51	0.5%	0.135	0.021
Other Race	7,251	3.1%	248	2.4%	<0.001	0.041	292	2.2%	248	2.4%	0.264	0.016
Unknown Race	21,548	9.2%	923	9.0%	0.518	0.007	1,284	9.6%	923	9.0%	0.680	0.006
Marital Status												
Married	90,945	38.8%	4,012	39.1%	0.488	0.007	5,138	38.4%	4,012	39.1%	0.252	0.016

Widowed	16,194	6.9%	859	8.4%	<0.001	0.055	954	7.1%	859	8.4%	0.706	0.005
Never Married	17,989	7.7%	748	7.3%	0.160	0.014	1,000	7.5%	748	7.3%	0.107	0.023
Divorced	9,207	3.9%	382	3.7%	0.304	0.010	417	3.1%	382	3.7%	0.853	0.003
Legally Separated	1,028	0.4%	31	0.3%	0.040	0.022	41	0.3%	31	0.3%	0.345	0.013
Domestic partner	585	0.2%	36	0.4%	0.045	0.019	21	0.2%	36	0.4%	0.203	0.018
Diagnosis												
Metabolic disorders	97,105	41.4%	3,630	35.4%	<0.001	0.124	5,217	39.0%	3,630	35.4%	0.008	0.037
Hypertensive diseases	98,698	42.1%	4,421	43.1%	0.040	0.021	6,253	46.7%	4,421	43.1%	<0.001	0.051
Diabetes mellitus	47,422	20.2%	1,882	18.3%	<0.001	0.047	2,781	20.8%	1,882	18.3%	0.003	0.042
Diseases of arteries, arterioles and capillaries	23,245	9.9%	829	8.1%	<0.001	0.064	1,124	8.4%	829	8.1%	0.013	0.035
Overweight, obesity and other hyperalimentation	25,489	10.9%	836	8.1%	<0.001	0.093	1,243	9.3%	836	8.1%	0.012	0.035
Cerebrovascular diseases	16,484	7.0%	660	6.4%	0.021	0.024	911	6.8%	660	6.4%	0.183	0.019
Other nutritional deficiencies	14,768	6.3%	451	4.4%	<0.001	0.084	685	5.1%	451	4.4%	0.049	0.027
Malnutrition	4,485	1.9%	120	1.2%	<0.001	0.060	164	1.2%	120	1.2%	0.074	0.025
Nicotine dependence, unspecified, uncomplicated	0	0.0%	0	0.0%	--	--	0	0.0%	0	0.0%	--	--
Alcohol related disorders	0	0.0%	0	0.0%	--	--	0	0.0%	0	0.0%	--	--
BMI	29.3 ± 6.1		30.2 ± 6.1		<0.001	0.146	30.0 ± 6.2		30.2 ± 6.1		0.031	0.054
0 - 18.50 kg/m2	3,382	1.4%	66	0.6%	<0.001	0.079	118	0.9%	66	0.6%	0.272	0.015
18.50 - 25 kg/m2	30,259	12.9%	723	7.0%	<0.001	0.196	984	7.3%	723	7.0%	0.458	0.010
25 - 30 kg/m2	53,721	22.9%	1,523	14.8%	<0.001	0.207	2,068	15.4%	1,523	14.8%	0.277	0.015
30 - 0 kg/m2	49,753	21.2%	1,650	16.1%	<0.001	0.132	2,338	17.5%	1,650	16.1%	0.359	0.013

Table S14. Demographic and clinical characteristics before and after matching between Cohort M and Cohort P.

Attribute	Before matching						After matching					
	Cohort M (tamsulosin)		Cohort P (alfuzosin)				Cohort M (tamsulosin)		Cohort P (alfuzosin)			
Patient numbers	N = 234,573		N = 5,456				N = 5,456		N = 5,456			
Demographics	N	%	N	%	p-value	SMD	N	%	N	%	p-value	SMD
Age												
Current Age	74.3 ± 11.4		71.0 ± 12.0		<0.001	0.281	77.0 ± 10.4		71.0 ± 12.0		0.310	0.019
Age at Index	67.6 ± 11.7		64.0 ± 11.8		<0.001	0.301	68.5 ± 10.8		64.0 ± 11.8		0.240	0.022
Ethnicity												
Not Hispanic or Latino	159,304	67.9%	3,443	63.1%	<0.001	0.101	8,984	67.1%	3,443	63.1%	0.330	0.019
Hispanic or Latino	14,927	6.4%	278	5.1%	<0.001	0.055	851	6.4%	278	5.1%	0.759	0.006
Unknown Ethnicity	60,342	25.7%	1,735	31.8%	<0.001	0.135	3,557	26.6%	1,735	31.8%	0.386	0.017
Race												
White	168,704	71.9%	4,016	73.6%	0.006	0.038	9,445	70.5%	4,016	73.6%	0.571	0.011
Asian	10,160	4.3%	191	3.5%	0.003	0.043	424	3.2%	191	3.5%	0.793	0.005
Black or African American	25,669	10.9%	390	7.1%	<0.001	0.133	1,883	14.1%	390	7.1%	0.970	0.001
American Indian or Alaska Native	476	0.2%	10	0.2%	0.750	0.004	33	0.2%	10	0.2%	1.000	<0.001
Native Hawaiian or Other Pacific Islander	765	0.3%	13	0.2%	0.259	0.017	31	0.2%	13	0.2%	0.705	0.007
Other Race	7,251	3.1%	184	3.4%	0.236	0.016	292	2.2%	184	3.4%	0.958	0.001
Unknown Race	21,548	9.2%	654	12.0%	<0.001	0.091	1,284	9.6%	654	12.0%	0.457	0.014
Marital Status												
Married	90,945	38.8%	2,251	41.3%	<0.001	0.051	5,138	38.4%	2,251	41.3%	0.938	0.001
Widowed	16,194	6.9%	211	3.9%	<0.001	0.135	954	7.1%	211	3.9%	0.580	0.011

Never Married	17,989	7.7%	262	4.8%	<0.001	0.119	1,000	7.5%	262	4.8%	0.929	0.002
Divorced	9,207	3.9%	153	2.8%	<0.001	0.062	417	3.1%	153	2.8%	0.166	0.027
Legally Separated	1,028	0.4%	14	0.3%	0.044	0.031	41	0.3%	14	0.3%	0.847	0.004
Domestic partner	585	0.2%	10	0.2%	0.332	0.014	21	0.2%	10	0.2%	1.000	<0.001
Diagnosis												
Metabolic disorders	97,105	41.4%	1,498	27.5%	<0.001	0.297	5,217	39.0%	1,498	27.5%	0.195	0.025
Hypertensive diseases	98,698	42.1%	1,376	25.2%	<0.001	0.363	6,253	46.7%	1,376	25.2%	0.877	0.003
Diabetes mellitus	47,422	20.2%	529	9.7%	<0.001	0.298	2,781	20.8%	529	9.7%	0.521	0.012
Diseases of arteries, arterioles and capillaries	23,245	9.9%	257	4.7%	<0.001	0.201	1,124	8.4%	257	4.7%	0.310	0.019
Overweight, obesity and other hyperalimentation	25,489	10.9%	361	6.6%	<0.001	0.151	1,243	9.3%	361	6.6%	0.727	0.007
Cerebrovascular diseases	16,484	7.0%	141	2.6%	<0.001	0.209	911	6.8%	141	2.6%	0.811	0.005
Other nutritional deficiencies	14,768	6.3%	247	4.5%	<0.001	0.078	685	5.1%	247	4.5%	0.642	0.009
Malnutrition	4,485	1.9%	14	0.3%	<0.001	0.160	164	1.2%	14	0.3%	0.548	0.012
Nicotine dependence, unspecified, uncomplicated	0	0.0%	0	0.0%	--	--	0	0.0%	0	0.0%	--	--
Alcohol related disorders	0	0.0%	0	0.0%	--	--	0	0.0%	0	0.0%	--	--
BMI	29.3 ± 6.1		28.8 ± 5.2		0.002	0.077	30.0 ± 6.2		28.8 ± 5.2		0.320	0.032
0 - 18.50 kg/m2	3,382	1.4%	58	1.1%	0.020	0.034	118	0.9%	58	1.1%	0.925	0.002
18.50 - 25 kg/m2	30,259	12.9%	537	9.8%	<0.001	0.096	984	7.3%	537	9.8%	0.215	0.024
25 - 30 kg/m2	53,721	22.9%	1,057	19.4%	<0.001	0.087	2,068	15.4%	1,057	19.4%	0.004	0.055
30 - 0 kg/m2	49,753	21.2%	778	14.3%	<0.001	0.183	2,338	17.5%	778	14.3%	0.202	0.024

Table S15. Demographic and clinical characteristics before and after matching between Cohort M and Cohort Q.

Attribute	Before matching						After matching					
	Cohort M (tamsulosin)		Cohort Q (silodosin)				Cohort M (tamsulosin)		Cohort Q (silodosin)			
Patient numbers	N = 234,573		N = 1,369				N = 1,369		N = 1,369			
Demographics	N	%	N	%	p-value	SMD	N	%	N	%	p-value	SMD
Age												
Current Age	74.3 ± 11.4		73.4 ± 11.0		0.005	0.077	77.0 ± 10.4		73.4 ± 11.0		0.759	0.012
Age at Index	67.6 ± 11.7		66.7 ± 11.5		0.005	0.076	68.5 ± 10.8		66.7 ± 11.5		0.890	0.005
Ethnicity												
Not Hispanic or Latino	159,304	67.9%	922	67.3%	0.656	0.012	8,984	67.1%	922	67.3%	1.000	<0.001
Hispanic or Latino	14,927	6.4%	71	5.2%	0.075	0.05	851	6.4%	71	5.2%	1.000	<0.001
Unknown Ethnicity	60,342	25.7%	376	27.5%	0.142	0.039	3,557	26.6%	376	27.5%	1.000	<0.001
Race												
White	168,704	71.9%	1,002	73.2%	0.296	0.029	9,445	70.5%	1,002	73.2%	0.408	0.032
Asian	10,160	4.3%	65	4.7%	0.450	0.020	424	3.2%	65	4.7%	0.928	0.003
Black or African American	25,669	10.9%	89	6.5%	<0.001	0.158	1,883	14.1%	89	6.5%	0.526	0.024
American Indian or Alaska Native	476	0.2%	10	0.7%	<0.001	0.077	33	0.2%	10	0.7%	1.000	<0.001
Native Hawaiian or Other Pacific Islander	765	0.3%	10	0.7%	0.009	0.056	31	0.2%	10	0.7%	0.002	0.121
Other Race	7,251	3.1%	41	3.0%	0.837	0.006	292	2.2%	41	3.0%	0.910	0.004
Unknown Race	21,548	9.2%	168	12.3%	<0.001	0.100	1,284	9.6%	168	12.3%	0.516	0.025
Marital Status												
Married	90,945	38.8%	653	47.7%	<0.001	0.181	5,138	38.4%	653	47.7%	0.730	0.013
Widowed	16,194	6.9%	62	4.5%	0.001	0.102	954	7.1%	62	4.5%	0.291	0.040

Never Married	17,989	7.7%	65	4.7%	<0.001	0.121	1,000	7.5%	65	4.7%	0.928	0.003
Divorced	9,207	3.9%	36	2.6%	0.014	0.073	417	3.1%	36	2.6%	0.715	0.014
Legally Separated	1,028	0.4%	10	0.7%	0.103	0.038	41	0.3%	10	0.7%	1.000	<0.001
Domestic partner	585	0.2%	10	0.7%	<0.001	0.069	21	0.2%	10	0.7%	1.000	<0.001
Diagnosis												
Metabolic disorders	97,105	41.4%	401	29.3%	<0.001	0.255	5,217	39.0%	401	29.3%	0.933	0.003
Hypertensive diseases	98,698	42.1%	385	28.1%	<0.001	0.296	6,253	46.7%	385	28.1%	1.000	<0.001
Diabetes mellitus	47,422	20.2%	165	12.1%	<0.001	0.223	2,781	20.8%	165	12.1%	0.593	0.020
Diseases of arteries, arterioles and capillaries	23,245	9.9%	76	5.6%	<0.001	0.164	1,124	8.4%	76	5.6%	0.623	0.019
Overweight, obesity and other hyperalimentation	25,489	10.9%	82	6.0%	<0.001	0.176	1,243	9.3%	82	6.0%	0.580	0.021
Cerebrovascular diseases	16,484	7.0%	74	5.4%	0.019	0.067	911	6.8%	74	5.4%	0.867	0.006
Other nutritional deficiencies	14,768	6.3%	69	5.0%	0.056	0.054	685	5.1%	69	5.0%	0.668	0.016
Malnutrition	4,485	1.9%	13	0.9%	0.009	0.081	164	1.2%	13	0.9%	0.463	0.028
Nicotine dependence, unspecified, uncomplicated	0	0.0%	0	0.0%	--	--	0	0.0%	0	0.0%	--	--
Alcohol related disorders	0	0.0%	0	0.0%	--	--	0	0.0%	0	0.0%	--	--
BMI	29.3 ± 6.1		28.8 ± 5.6		0.061	0.086	30.0 ± 6.2		28.8 ± 5.6		0.481	0.044
0 - 18.50 kg/m2	3,382	1.4%	16	1.2%	0.398	0.024	118	0.9%	16	1.2%	0.713	0.014
18.50 - 25 kg/m2	30,259	12.9%	147	10.7%	0.017	0.067	984	7.3%	147	10.7%	1.000	<0.001
25 - 30 kg/m2	53,721	22.9%	269	19.6%	0.004	0.080	2,068	15.4%	269	19.6%	0.699	0.015
30 - 0 kg/m2	49,753	21.2%	210	15.3%	<0.001	0.152	2,338	17.5%	210	15.3%	0.712	0.014

Table S16. Demographic and clinical characteristics before and after matching between Cohort M and Cohort R.

Attribute	Before matching						After matching					
	Cohort M (tamsulosin)		Cohort R (prazosin)				Cohort M (tamsulosin)		Cohort R (prazosin)			
Patient numbers	N = 234,573		N = 609				N = 609		N = 609			
Demographics	N	%	N	%	p-value	SMD	N	%	N	%	p-value	SMD
Age												
Current Age	74.3 ± 11.4		68.7 ± 16.7		<0.001	0.390	77.0 ± 10.4		68.7 ± 16.7		0.731	0.02
Age at Index	67.6 ± 11.7		61.3 ± 16.3		<0.001	0.440	68.5 ± 10.8		61.3 ± 16.3		0.679	0.024
Ethnicity												
Not Hispanic or Latino	159,304	67.9%	426	70.0%	0.282	0.044	8,984	67.1%	426	70.0%	0.900	0.007
Hispanic or Latino	14,927	6.4%	37	6.1%	0.771	0.012	851	6.4%	37	6.1%	0.199	0.074
Unknown Ethnicity	60,342	25.7%	146	24.0%	0.324	0.041	3,557	26.6%	146	24.0%	0.595	0.030
Race												
White	168,704	71.9%	422	69.3%	0.150	0.058	9,445	70.5%	422	69.3%	0.579	0.032
Asian	10,160	4.3%	25	4.1%	0.784	0.011	424	3.2%	25	4.1%	0.548	0.034
Black or African American	25,669	10.9%	99	16.3%	<0.001	0.155	1,883	14.1%	99	16.3%	0.592	0.031
American Indian or Alaska Native	476	0.2%	10	1.6%	<0.001	0.151	33	0.2%	10	1.6%	0.001	0.183
Native Hawaiian or Other Pacific Islander	765	0.3%	10	1.6%	<0.001	0.134	31	0.2%	10	1.6%	1.000	<0.001
Other Race	7,251	3.1%	19	3.1%	0.967	0.002	292	2.2%	19	3.1%	0.634	0.027
Unknown Race	21,548	9.2%	41	6.7%	0.036	0.091	1,284	9.6%	41	6.7%	0.578	0.032
Marital Status												
Married	90,945	38.8%	195	32.0%	0.001	0.142	5,138	38.4%	195	32.0%	0.051	0.112

Widowed	16,194	6.9%	36	5.9%	0.335	0.041	954	7.1%	36	5.9%	0.530	0.036
Never Married	17,989	7.7%	81	13.3%	<0.001	0.185	1,000	7.5%	81	13.3%	0.126	0.088
Divorced	9,207	3.9%	27	4.4%	0.519	0.025	417	3.1%	27	4.4%	0.668	0.025
Legally Separated	1,028	0.4%	10	1.6%	<0.001	0.119	41	0.3%	10	1.6%	1.000	<0.001
Domestic partner	585	0.2%	10	1.6%	<0.001	0.144	21	0.2%	10	1.6%	1.000	<0.001
Diagnosis												
Metabolic disorders	97,105	41.4%	212	34.8%	0.001	0.136	5,217	39.0%	212	34.8%	0.331	0.056
Hypertensive diseases	98,698	42.1%	257	42.2%	0.950	0.003	6,253	46.7%	257	42.2%	0.382	0.050
Diabetes mellitus	47,422	20.2%	108	17.7%	0.128	0.063	2,781	20.8%	108	17.7%	1.000	<0.001
Diseases of arteries, arterioles and capillaries	23,245	9.9%	61	10.0%	0.930	0.004	1,124	8.4%	61	10.0%	0.639	0.027
Overweight, obesity and other hyperalimentation	25,489	10.9%	68	11.2%	0.812	0.010	1,243	9.3%	68	11.2%	0.787	0.015
Cerebrovascular diseases	16,484	7.0%	40	6.6%	0.658	0.018	911	6.8%	40	6.6%	1.000	<0.001
Other nutritional deficiencies	14,768	6.3%	31	5.1%	0.221	0.052	685	5.1%	31	5.1%	0.689	0.023
Malnutrition	4,485	1.9%	10	1.6%	0.627	0.020	164	1.2%	10	1.6%	0.528	0.036
Nicotine dependence, unspecified, uncomplicated	0	0.0%	0	0.0%	--	--	0	0.0%	0	0.0%	--	--
Alcohol related disorders	0	0.0%	0	0.0%	--	--	0	0.0%	0	0.0%	--	--
BMI	29.3 ± 6.1		30.3 ± 6.3		0.018	0.160	30.0 ± 6.2		30.3 ± 6.3		0.964	0.004
0 - 18.50 kg/m2	3,382	1.4%	10	1.6%	0.679	0.016	118	0.9%	10	1.6%	1.000	<0.001
18.50 - 25 kg/m2	30,259	12.9%	53	8.7%	0.002	0.136	984	7.3%	53	8.7%	0.178	0.077
25 - 30 kg/m2	53,721	22.9%	100	16.4%	<0.001	0.164	2,068	15.4%	100	16.4%	0.384	0.050
30 - 0 kg/m2	49,753	21.2%	114	18.7%	0.133	0.062	2,338	17.5%	114	18.7%	0.711	0.021
